# Supplementary material for: An optogenetic-phosphoproteomic study reveals dynamic Akt1 signaling profiles in endothelial cells
Source: Nat Commun. 2023 Jun 26;14:3803. doi: 10.1038/s41467-023-39514-1 (PMC10293293; doi:10.1038/s41467-023-39514-1)
Supplement: Supplementary file 1 — Supplementary Information [file 41467_2023_39514_MOESM1_ESM.pdf]

Supplementary Information for

An Optogenetic-Phosphoproteomic Study Reveals Dynamic Akt1 Signaling Profiles in Endothelial Cells

Wenping Zhou[1,2,3*], Wenxue Li[1,4*], Shisheng Wang[5*], Barbora Salovska[1,4], Zhenyi Hu[1,4], Bo Tao[1,3], Yi Di[1,4], Ujwal Punyamurtula[6], Benjamin E. Turk[1], William C. Sessa[1,3†], Yansheng Liu[1,4†]

[†] **Corresponding authors:**

william.sessa@yale.edu

yansheng.liu@yale.edu

The PDF file includes

Supplementary Table 1

Supplementary Figure 1 to 12

Supplementary Table 1. Information of reagents and materials

REAGENT or RESOURCE	SOURCE	IDENTIFIER (& Dilution ratio for antibodies)
Antibodies		
monoclonal rabbit phospho-Akt T308	cell signaling	Cat#2965; 1:1000 dilution
monoclonal rabbit phospho-Akt S473	cell signaling	Cat#9271; 1:1000 dilution
monoclonal mouse pan-Akt	cell signaling	Cat#2920; 1:1000 dilution
chicken polyclonal anti-GFP	Abcam	Cat#ab13970; 1:1000 dilution
monoclonal rabbit phospho-eNOS S1177	cell signaling	Cat#9570 ; 1:1000 dilution
polyclonal rabbit eNOS	cell signaling	Cat#9572; 1:1000 dilution
monoclonal rabbit phospho-Erk T202/Tyr204.	cell signaling	Cat#4370 ; 1:1000 dilution
monoclonal rabbit Erk	cell signaling	Cat#4695; 1:1000 dilution
polyclonal rabbit phospho-GSK-3β S9	cell signaling	Cat#9336; 1:1000 dilution
monoclonal rabbit GSK	cell signaling	Cat#9315; 1:1000 dilution
Polyclonal Rabbit Phospho-NDRG1 Ser330	cell signaling	Cat#3506; 1:1000 dilution
Polyclonal Rabbit NDRG1	cell signaling	Cat#5196; 1:1000 dilution
Polyclonal Rabbit Phospho-NEDD4L Ser448	cell signaling	Cat#8063; 1:1000 dilution
Polyclonal Rabbit NEDD4L Antibody Cell signaling	cell signaling	Cat#4013; 1:1000 dilution
monoclonal mouse Hsp90	BD Biosciences	Cat# 610419; 1:1000 dilution
monoclonal mouse β-Actin	Sigma-Aldrich	Cat# A5441; 1:1000 dilution
goat anti-rabbit Alexa Fluor 680	Thermo Fisher	Cat#A20984; 1:1000 dilution
goat anti-chicken Alexa Fluor 488	Thermo Fisher	Cat#A11039; 1:1000 dilution
goat anti-mouse Alexa Fluor 680	Thermo Fisher	Cat#A21057; 1:1000 dilution
goat anti-mouse 800	Rockland	Cat#610-145-002-0.5; 1:1000 dilution
Donkey anti-rabbit IgG-HRP-linked whole Ab	Cytiva	NA934; 1:1000 dilution
Sheep anti-mouse IgG-HRP-linked whole Ab	Cytiva	NA931; 1:1000 dilution
Bacterial and virus strains		
DB3.1 cell strain	Invitrogen/ Thermo Fisher	Cat#11782-018
Chemicals, Peptides, and Recombinant proteins		
optiMEM media	Thermo Fisher	Cat#31985062
Lipofectamine 2000	Invitrogen/Thermo Fisher	Cat#11668019
Medium 199	Thermo Fisher	Cat# 11150067
Medium 199, no phenol red	Thermo Fisher	Cat# 11043023
DMEM	Thermo Fisher	Cat# 11965092
DMEM, no phenol red	Thermo Fisher	Cat# 21063029
HEPES	Thermo Fisher	Cat# 15630080
Halt™ Protease and Phosphatase Inhibitor Cocktail	Thermo Fisher	Cat# 78440

Recombinant Human Angiopoietin-1 Protein	R&D systems	Cat# 923-AN
cOmplete Protease Inhibitor Cocktail	Roche/Sigma-Aldrich	Cat# 11697498001
Pefabloc SC AEBSF	Roche/Sigma-Aldrich	Cat# 11429868001
sepiapterin	Sigma- Aldrich	Cat# 17094-01-8
LY294002	Sigma- Aldrich	Cat# 934389-88-5
ionomycin		Cat# 3642126
High-Select™ Fe-NTA Phosphopeptide Enrichment Kit	Thermo Fisher	Cat# A32992
FSBA	Sigma- Aldrich	Cat #F9128
Recombinant AKT1	Sigma- Aldrich	Cat #SRP0353
ATP	Sigma- Aldrich	Cat #20306
Experimental models: cell lines		
EA.hy926	ATCC	Cat# CRL-2922
HEK 293T	ATCC	Cat# CRL-3216
HeLa CCL-2	ATCC	Cat# CCL-2
HUVEC	Yale Vascular Biology & Therapeutics (VBT) Program	Item number T25
EA.hy926 mCherry-CYR2-Akt1 and GFP-CIBN-CAAX	This study	N/A
HUVEC mCherry-CYR2-Akt1 and GFP-CIBN-CAAX	This study	N/A
Recombinant DNA		
mCherry-CYR2-Akt1	Ref [1]	N/A
GFP-CIBN-CAAX	Ref [2]	N/A
Software and Algorithms		
Image Studio software	Li-COR	https://www.licor.com/bio/image-studio-lite/download
Image J	NIH	https://imagej.nih.gov/ij/
R Software	N/A	https://www.r-project.org/
Velocity Software	Velocity	https://www.volocity4d.com/

Supplementary Figure 1

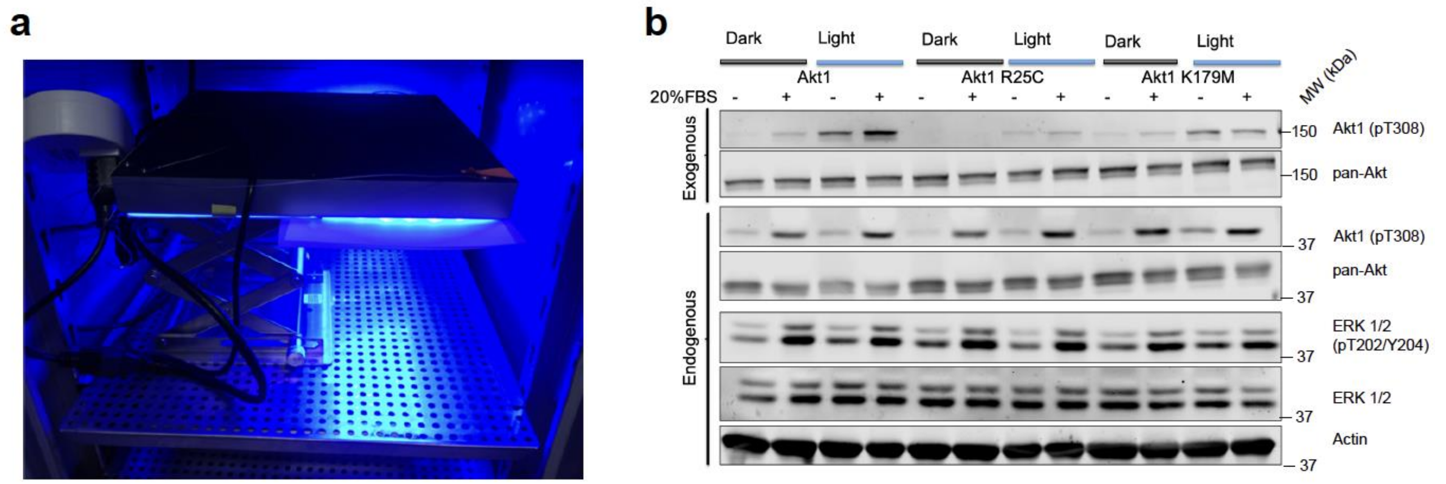

Figure S1. Establishment of the optogenetic cell culture system for inducing and controlling Akt phosphorylation. **(a)** A manually engineered light system in cell culture incubator, which has a blue LED light (460-470 nm) plugged into a timer, a lifting table, a filter and a diffuser between the light and the sample to achieve proper and uniform light exposure. The timer can control the light exposure time from 1 minute to 24 hours and set programs to achieve periodic light illumination. The lifting table can change the distance between the light and the sample to adjust the light magnitude reached the sample. **(b)** Immunoblot comparing phosphorylation of exogenous optogenetic wild-type Akt1 and Akt1 R25C and Akt1 K179M mutants in response to20% FBS or blue light illumination. Akt1 R25C carried a mutation in the PH domain that blocks recruitment to the plasma membrane, preventing phosphorylation and or further activation. Akt1 K179M has a kinase inactivating mutation in the catalytic domain. The immunoblot shows that the optogenetic wild-type Akt1 could be phosphorylated by serum or blue light stimulation, while Akt1 R25C was weakly phosphorylated. Kinase inactive Akt1 K179M was phosphorylated to a lower extent than wild-type Akt1. Endogenous Akt1 was phosphorylated following serum stimulation regardless of the light condition.

Supplementary Figure 2

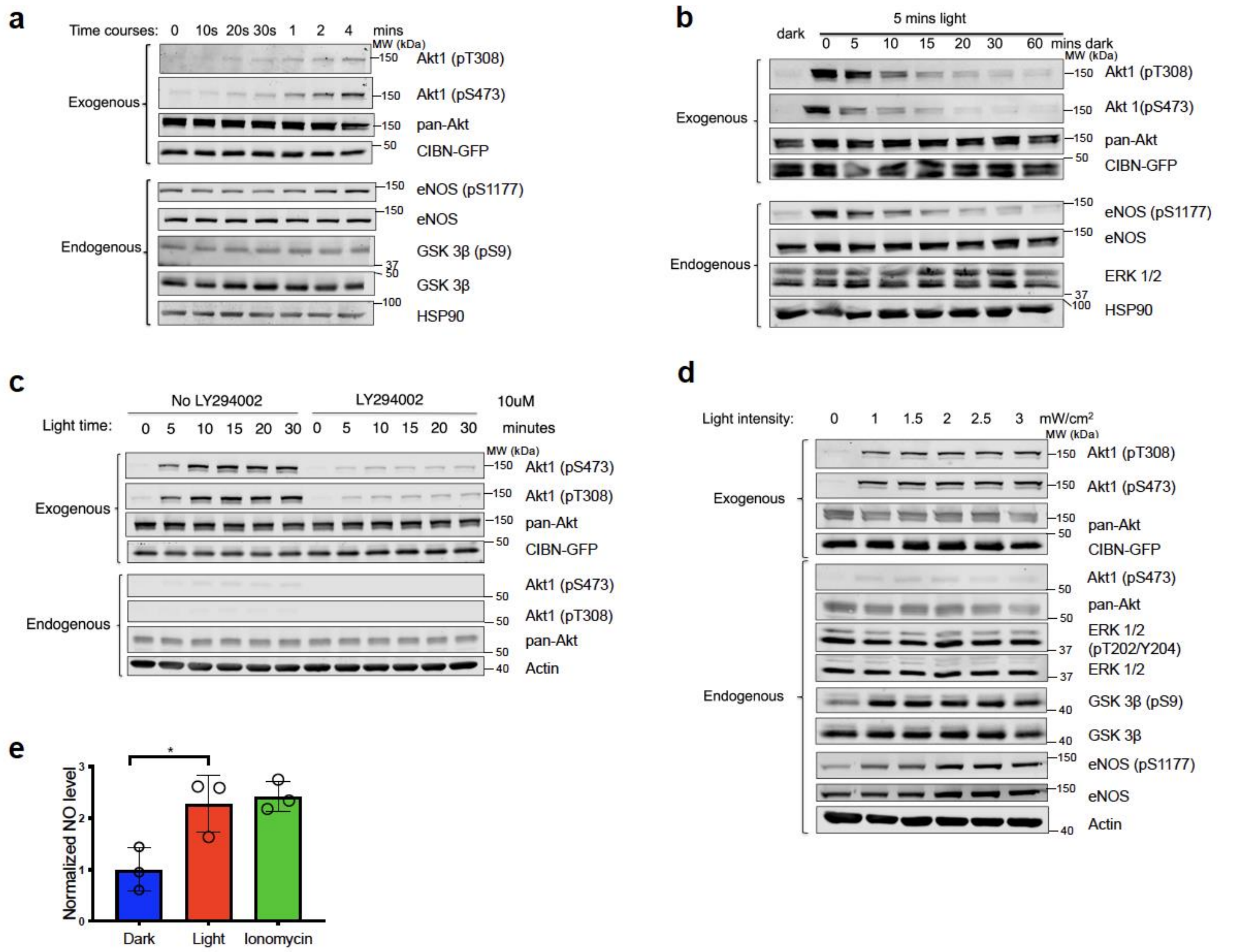

Figure S2. Characterization of the light activated Akt system.

(a) Western blot of infected EA.hy 926 with 0.25 mW/cm2 light with different time courses**. (b)** Western blot of 5-minute light illumination followed by 5-60 minutes dark in induced EA.hy 926 (Light intensity,16 mW/cm2). **(c)** Western blot of LY294002 (10 μM) treated EA.hy 926 cells compared with control DMSO treatment showing phosphorylated-Akt and total Akt levels. **(d)** Western blot of phosphorylated Akt, total Akt, Akt substrates including GSK and eNOS and independent Erk signaling with light stimuli from 1 mW/cm2 to 3 mW/cm2 with interval 0.5 light intensity increase. **(e)** Nitric oxide (NO) production assay comparing the light-induced Akt and positive control with ionomycin (1 uM) treatment in HUVECs (n=3 biologically independent cell samples). Error bar indicates SD. For (**a-d**), two times of WB experiments were repeated independently with similar results.

Supplementary Figure 3

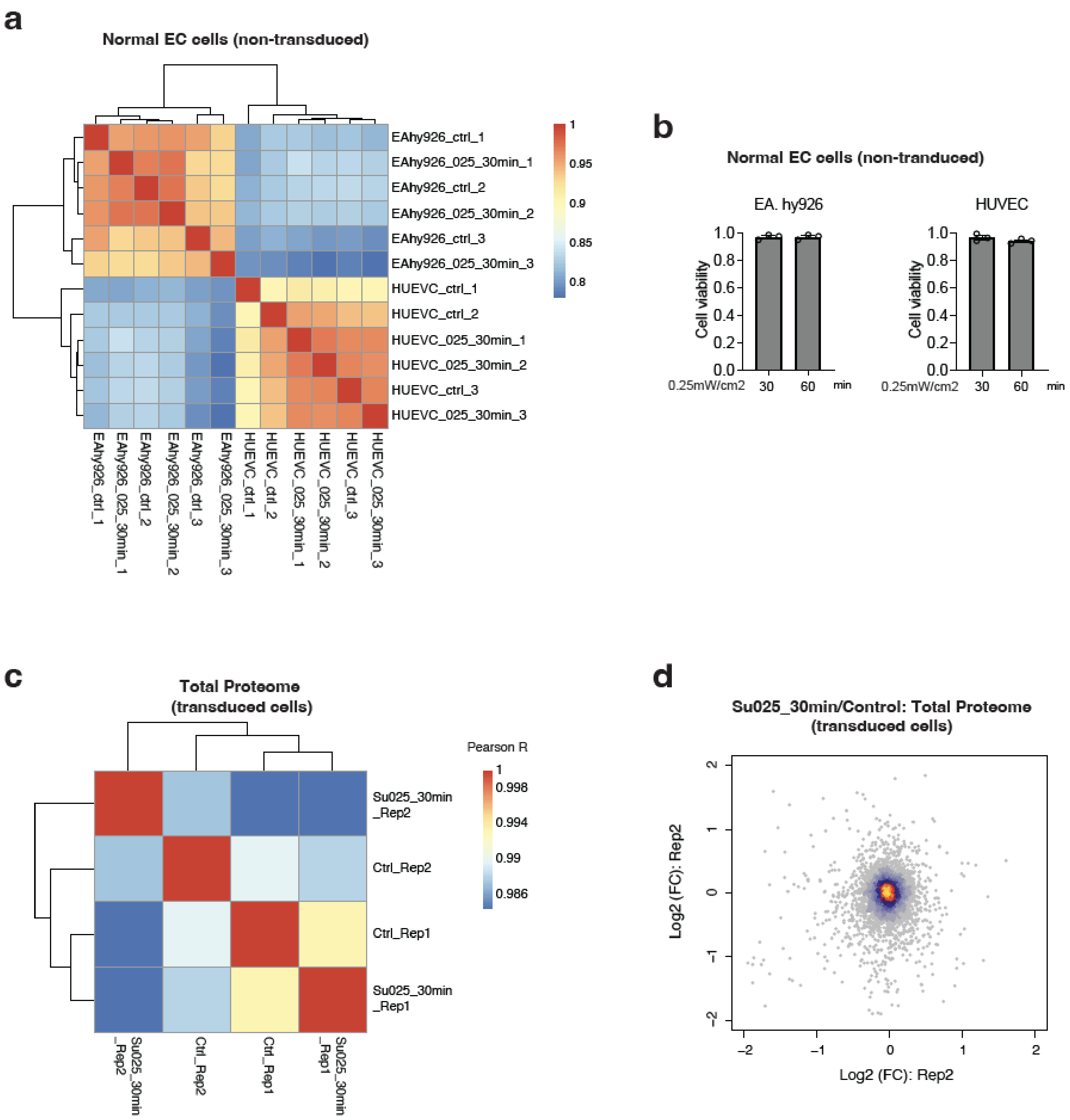

Figure S3. Additional quality controls and considerations for the OptopDIA design. (a-b) The normal (i.e., non-transduced) EA.hy 926 and HUVEC endothelial cells were both stimulated with 0.25 mW/cm^2 blue light for 30 mins and subjected to the total phosphoproteomic measurement. **(a)** The heatmap based the hierarchical clustering analysis (HCA) did not show detectable phosphoproteomic variance and any cell viability difference, as compared to the biological replicates. **(b)** Both normal EC cell lines were stained trypan blue at 30 and 60 mins light stimulation of 0.25 mW/cm^2 and the countered using TC20 automated cell counter, suggesting almost no cells were dead (n=3 biologically independent cell samples). Error bar indicates SEM. **(c-d)** The total proteome regulated by the 0.25 mW/cm^2 blue light for 30 mins in the transduced EA.hy 926 cells. **(c)** HCA indicated no detachable proteome alteration induced by light which is similar to the scenario of biological replicates. **(d)** No correlation was observed for light induced proteome changes in 30 mins between biological replicates (x and y axis). FC means the fold-change of light simulation versus control.

Supplementary Figure 4

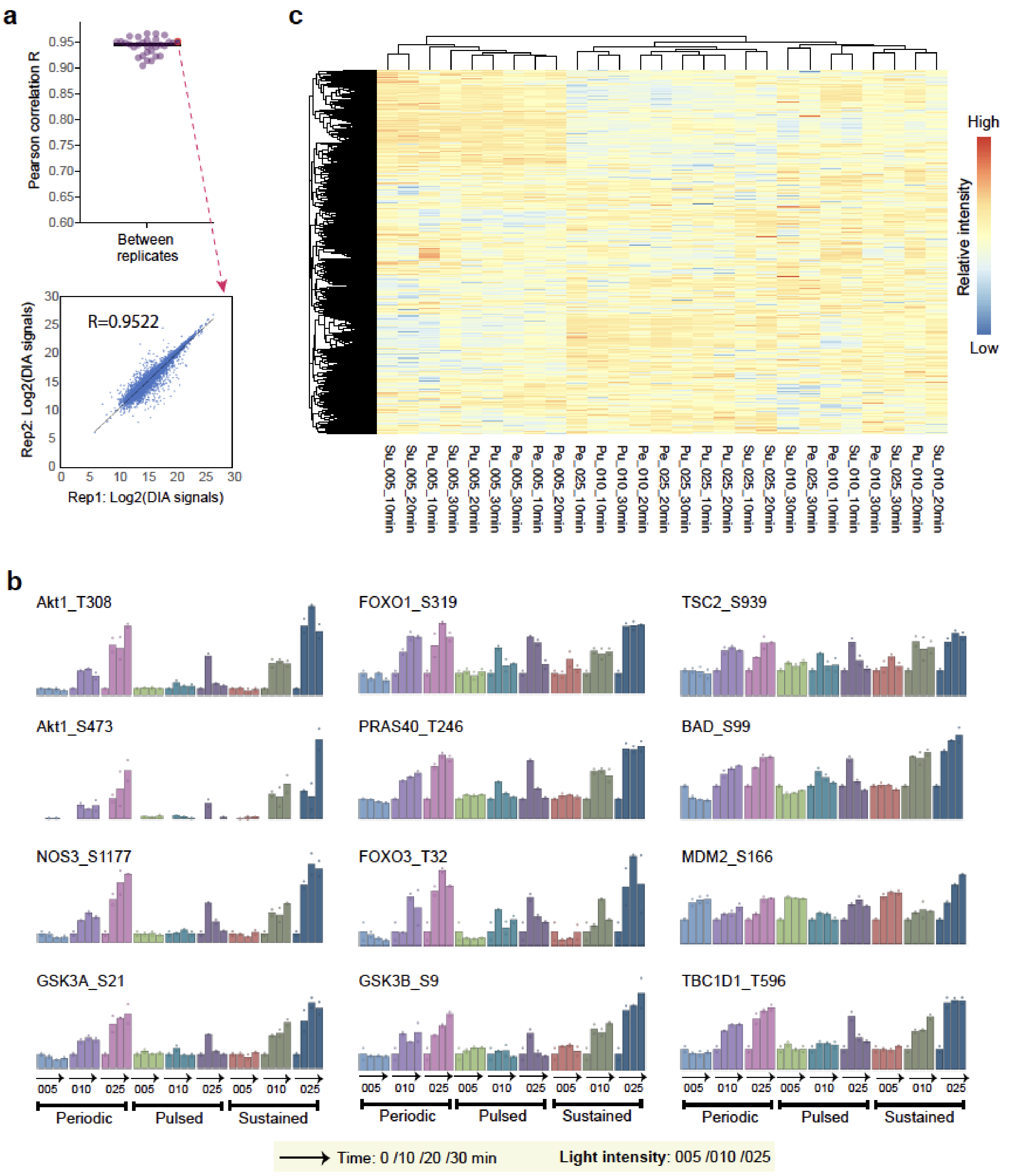

Figure S4. Qualifying the dataset generated by Optop-DIA workflow.
(a) The *Pearson* correlation coefficients achieved by all biological replicates with the example close to the averaged level visualized by scatterplot below. **(b)** Individual examples of Akt substrate P-sites quantified across activation conditions. **(c)** Hierarchical clustering analysis (HCA) on all the conditions (n=2 biologically independent samples). Error bar denotes SD. histogram bars denote Mean.

Supplementary Figure 5

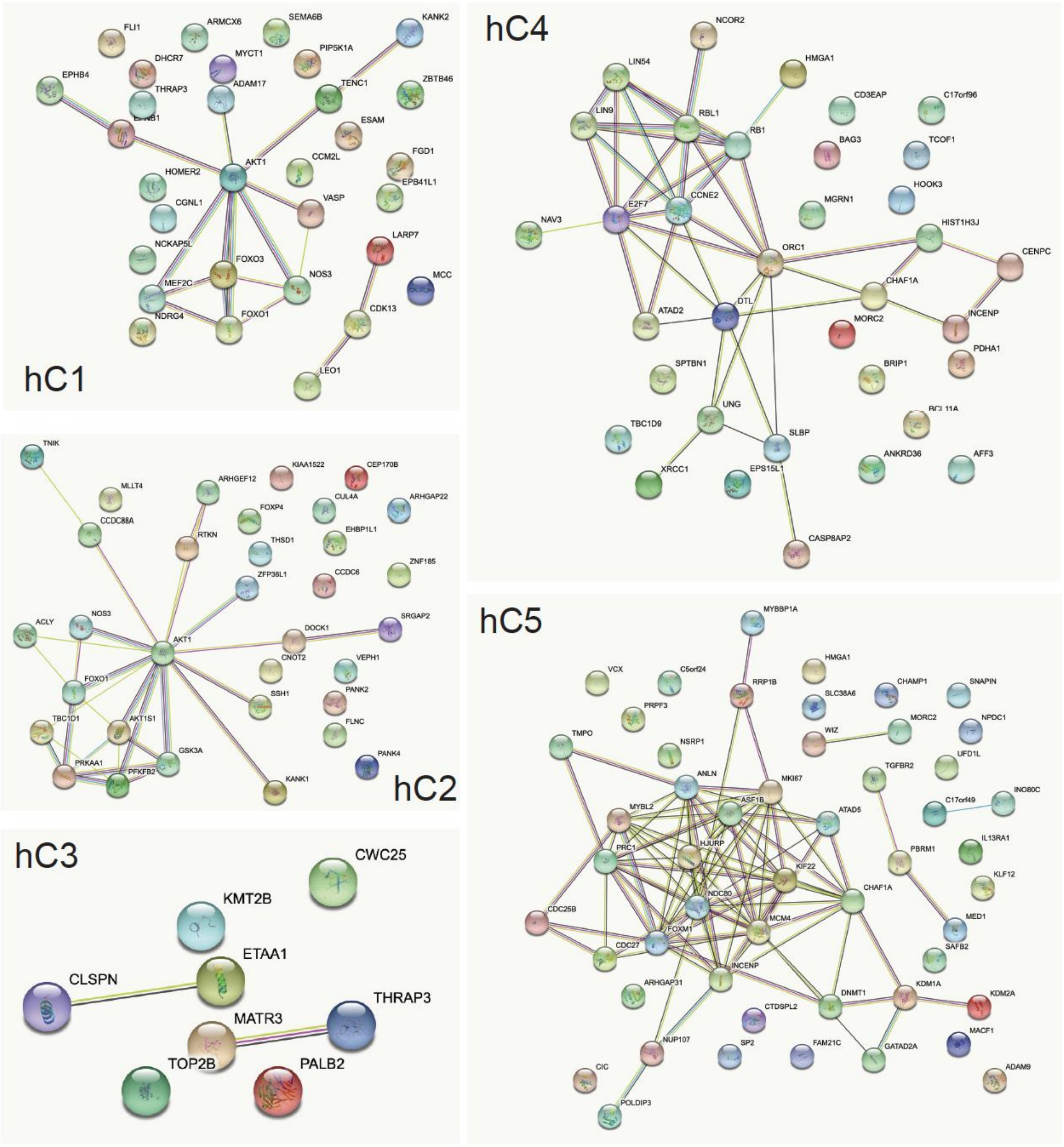

Figure S5. The protein names and their potential protein-protein interactions (PPIs) corresponding to the P-sites in hC1-hC5 (Re. Figure 2).

The protein names were submitted to STRING (https://string-db.org/) which reports the potential PPI networks for hC1-hC5 respectively.

Supplementary Figure 6

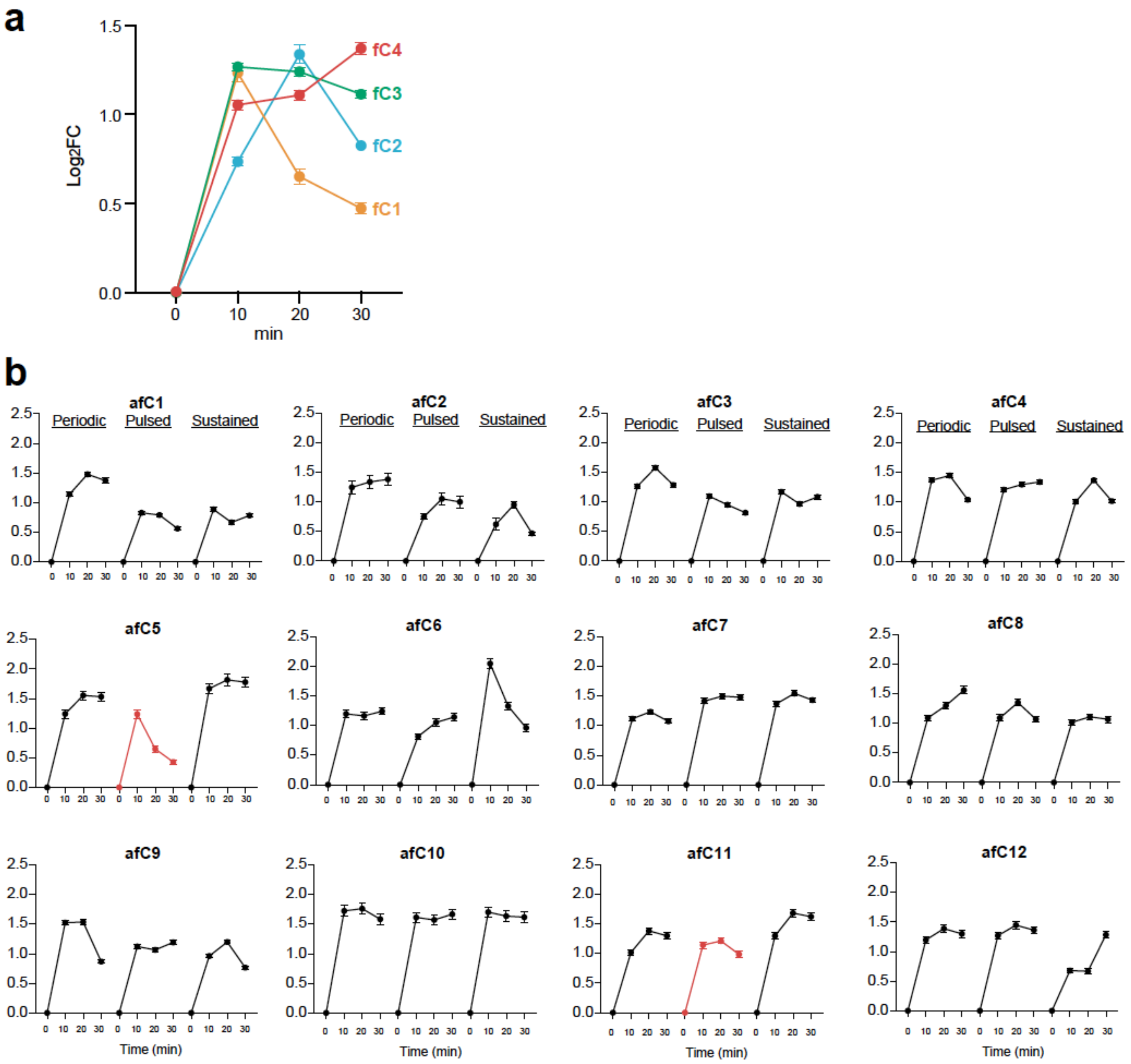

Figure S6. The averaged profiles for Clusters fC1-fC4 and afC1-afC12. (a) The mean and SEM (shown as error bars) of log2 Fold changes values in each time point (to 0 mins) for Clusters fC1-fC4 (re.Figure 3B, n=189, 247 838, and 599 observations of P-sites in fC1-fC4 curves). **(b)** The mean and SEM (shown as error bars) of log2 Fold changes values in each time point (to 0 mins) for Clusters afC1-afC12 (re. Figure 4, n=119, 95, 181, 203, 93, 85, 157, 128, 163, 195, 118, 98 observations of P-sites in afC1-afC12 lists). The red curve denotes the expected pattern of Akt direct substrates in the pulsed condition (the enrichment percentage of P-sites with strict Akt motif is 30.11% and 12.71% for afC5 and afC11).

Supplementary Figure 7

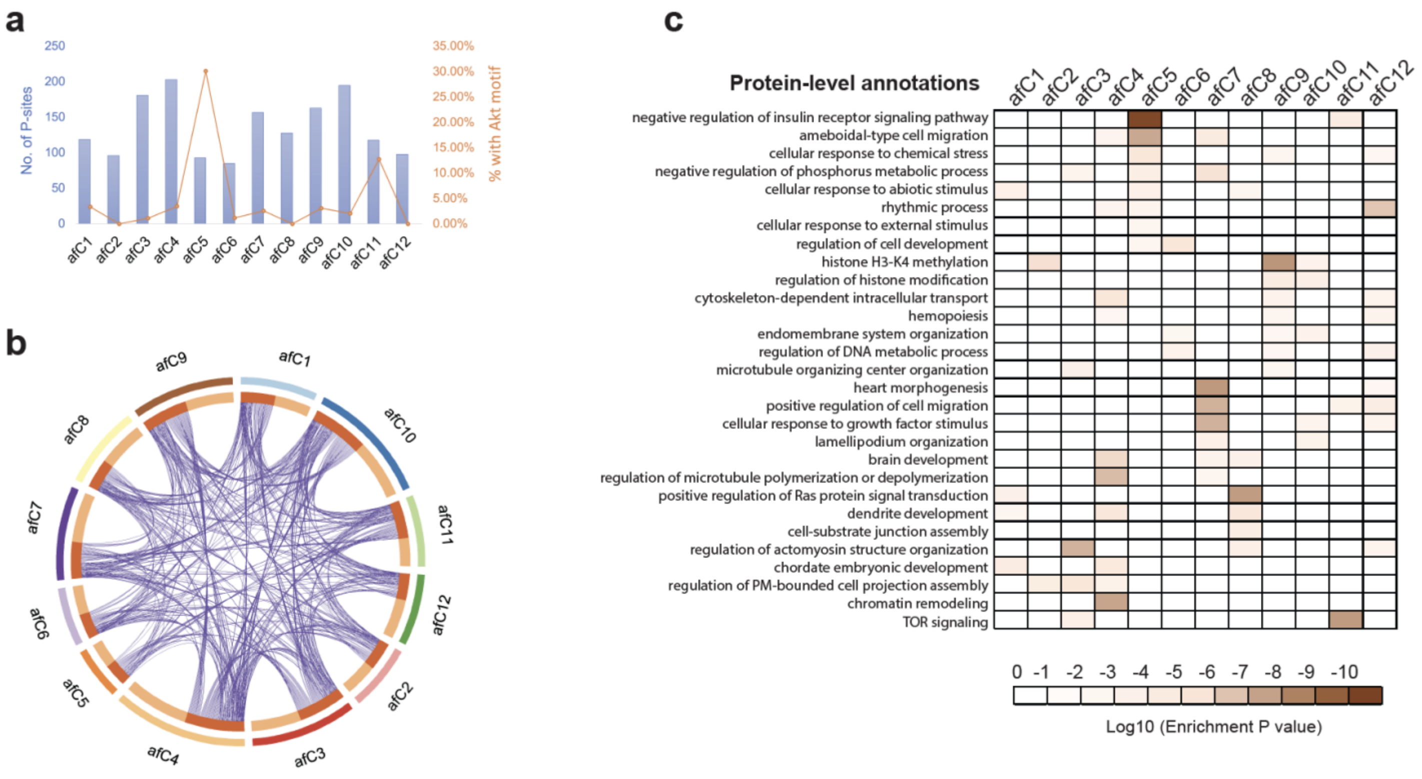

Figure S7. P sites and their corresponding protein annotations in each of the 12 FCM clusters.
(a) Numbers of P-sites of afC1-afC12 was shown in histograms, with the secondary axis (i.e., the orange line) showing different percentages of Akt strict motifs in afC1-afC12. **(b)** The overlapping proteins between any two clusters among afC1-afC12 are linked by a purple line. Note this circos plot generated by Metascape [3] visualizes the fact that despite P-sites are distinctive between afC1-afC12, the protein names can be overlapping extensively, which, in turn, makes the protein-level annotation less-useful than P-site level analysis. **(c)** Representative GO biological processes (BPs) enriched from proteins corresponding to P-sites in afC1-afC12. This protein-level enrichment analysis was performed by Metascape. The enrichment *P* values are reported by *hypergeometric* test using Metascape (without adjustment, please see https://metascape.org/blog/?p=122).

Supplementary Figure 8

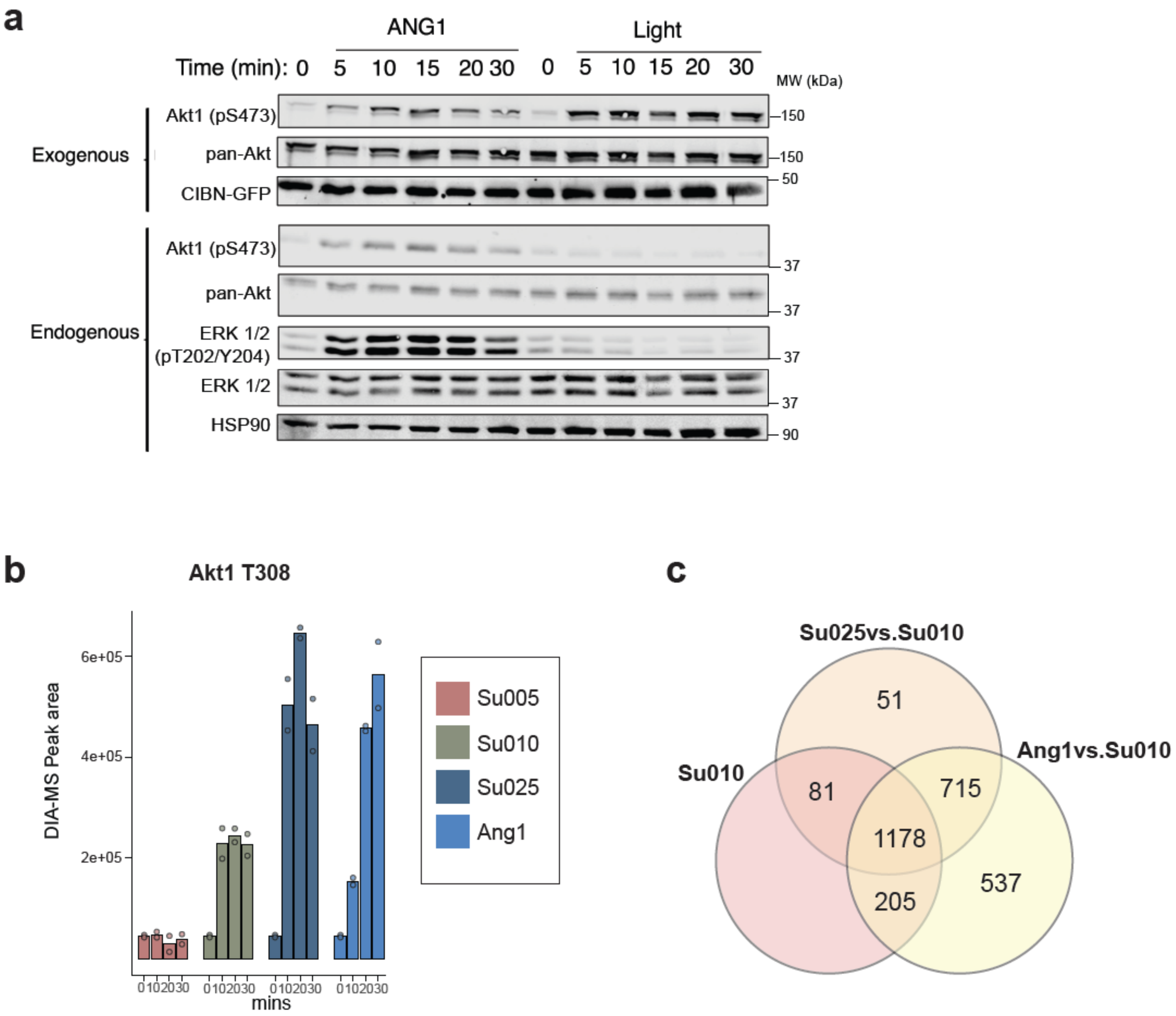

Figure S8. The basic considerations for comparing Angiopoietin and Akt signaling in EC cells.
(a) Western blot validating the systemic graph that light stimuli (LED light, 16 mW/cm2) can induce Akt phosphorylation compared with growth factor stimuli Ang1 (400 ng/ul) in infected HUVECs at P5. **(b)** The Optop-DIA result in EA.hy 926 cells with Angiopoietin-1 (Ang1) stimulation for 10, 20, and 30mins, as compared to the Sustained (Su) activation with different Akt intensity (n=2 biologically independent samples). Note that Akt p-T308 induced by Ang1 represents the phosphorylation extent *between* Su010 and Su025 conditions, setting a nice comparable basis. Center bars denote Mean. **(c)** Venn diagram as an output from MaSigPro analysis, which denotes 2,635 P-sites showing differential temporal profiles (DTPs) between Su010, Su025, and Ang1 experiments (BH adjusted q<0.05). For (**a**), two times of WB experiments were repeated independently with similar results.

Supplementary Figure 9

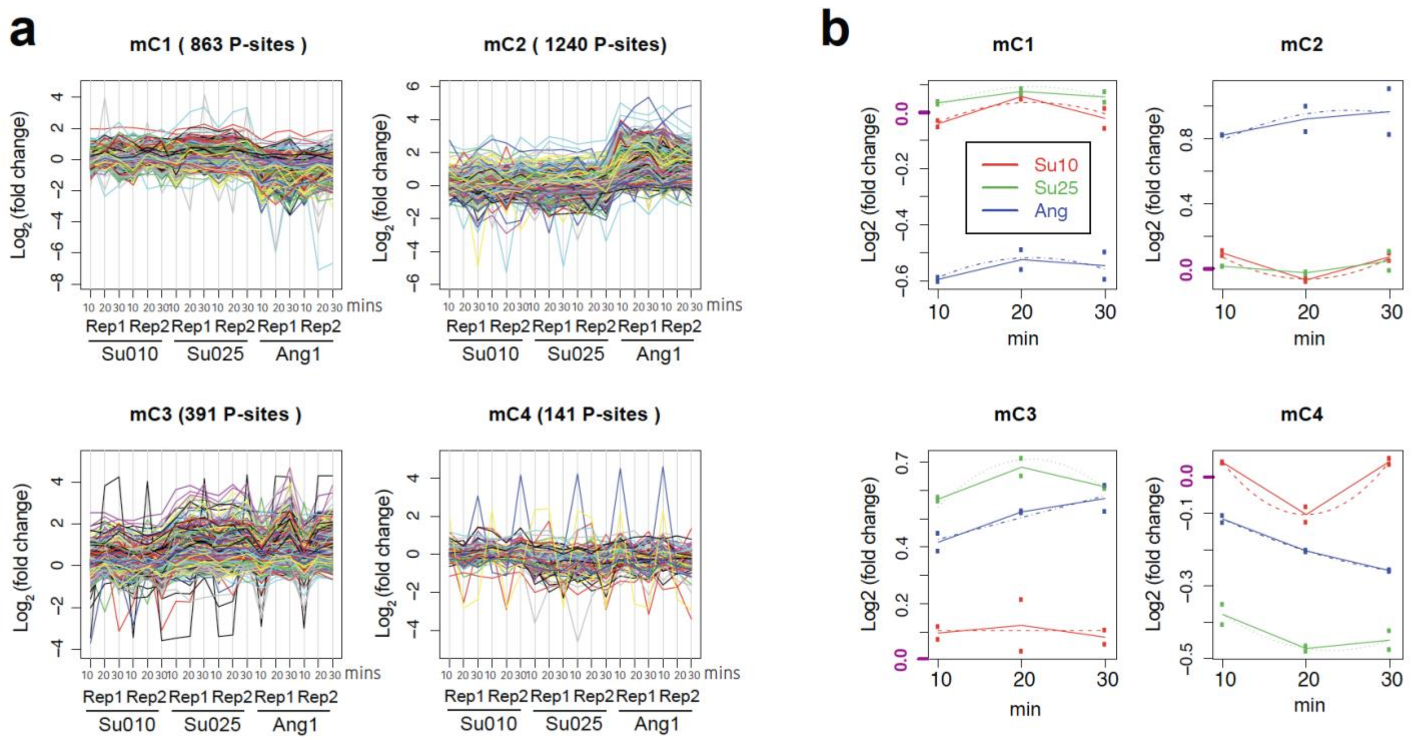

Figure S9 The maSigPro analysis and plot for comparing the the Light- and Ang1- activated signaling. (**a**) maSigPro classified differential temporal profiles from time-course datasets into four clusters (mC1-mC4). Please also see Figure S8. The log2 fold changes (FC) values in each time point (to 0 mins) were shown for three conditions and two biological replicates **(b)** The averaged Log2- fold change profiles of P-site in mC1-mC4 clusters. Two biological replicates were shown. Dots show actual fold-change values. Solid lines denote the average value of fold-change at each time point for each experimental group. Fitted curves after computed regression analysis using maSigPro[4] are displayed as dotted lines.

Supplementary Figure 10

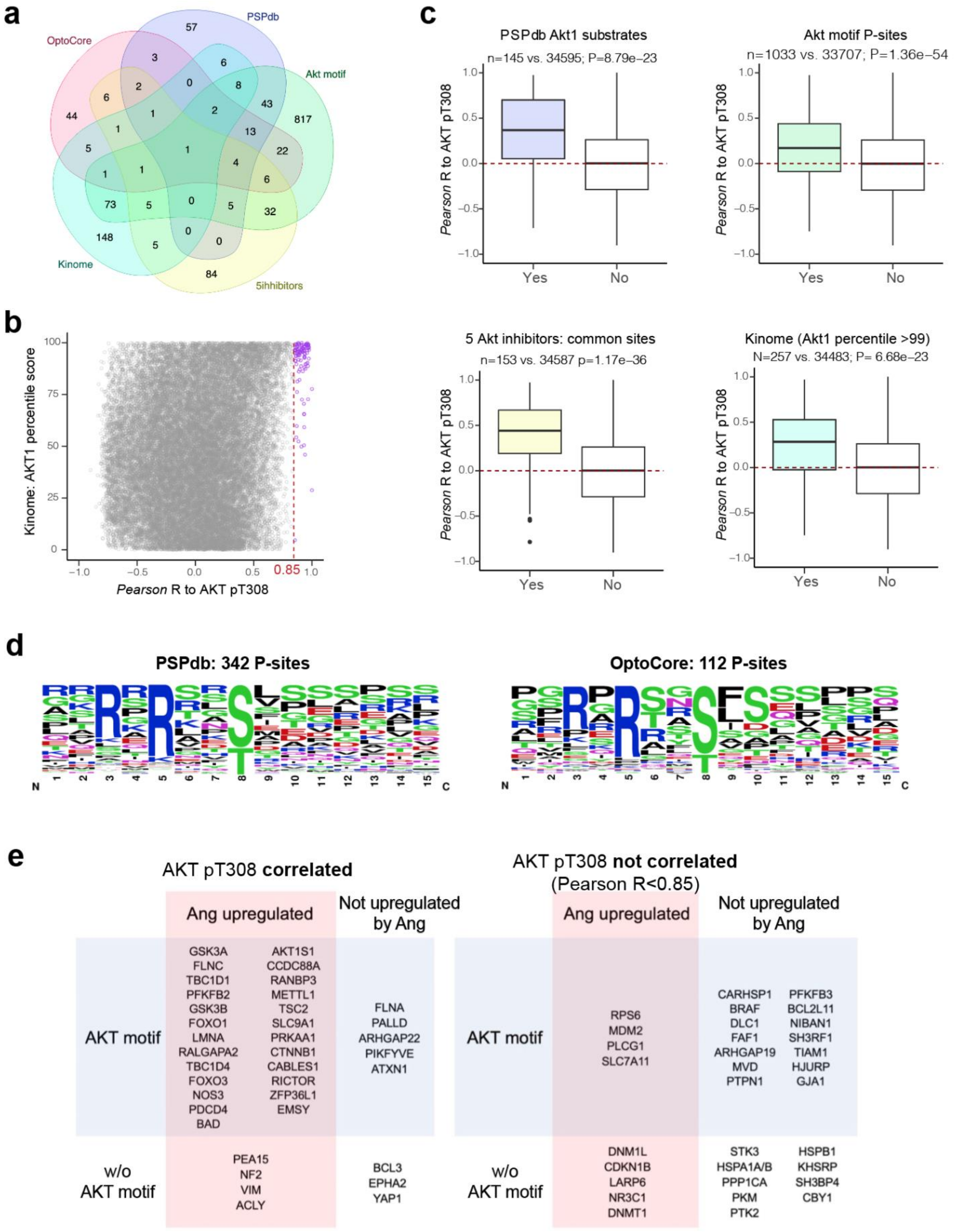

Figure S10. Bioinoformatic validation of Akt co-varying P-sites (a) The phosphosite level overlapping analysis between the five lists i.e., OptoCore, PsPdb, and Akt motif, 5 inhibitors and kinome (Please see the description in the Results). Note each list has significantly fraction of P sites not covered by other lists. **(b)** The quantitative Pearson correlation of each phosphosite to Akt pT308 as compared to the kinome percentile score. Note all OptoCore sites (R>0.85 to Akt pT308) are labeled in purple circles and had a much higher kinome percentile score. **(c)** Those P sites listed in PsPdb, and Akt motif, 5 inhibitors and kinome were identified by OptopDIA with much higher Pearson correlation R, compared to other P sites. P values are calculated by the two-sided Wilcox test. Box-plot elements: center line, median; box limits, first and third quartiles; whiskers, 1.5*interquartile range; outlying points, data beyond the 1.5 interquartile range. **(d)** The globally similar distribution frequency of amino acids surrounding the phosphorylated S/Tfor all PSPdb contained Akt substrate P-sites and OptoCore P sites. **(e)** An extended comparison between P-sites correlating (R > 0.5) with AKT1-Thr308 phosphorylation and Johnson et al. [5] using the scoring at >94.5% based on peptide library analysis. Most substrates phosphorylated in response to Ang also correlate with AKT1-Thr308 phosphorylation well across all conditions (directly or indirectly) by opto-AKT. Notably, this set includes a number of well-characterized substrates that do not localize to the cell periphery – FOXO1/3, BAD, TBC1D4 (aka AS160), and AKT1S1 (PRAS40) among them. Most reported substrates that did not correlate with AKT1-pT308 likewise did not become phosphorylated in response to Ang1. Interestingly, two “Ang only” substrates localize to the plasma membrane (PLC-γ, SLC7A11/xCT), but none localize exclusively to endomembranes.

Supplementary Figure 11

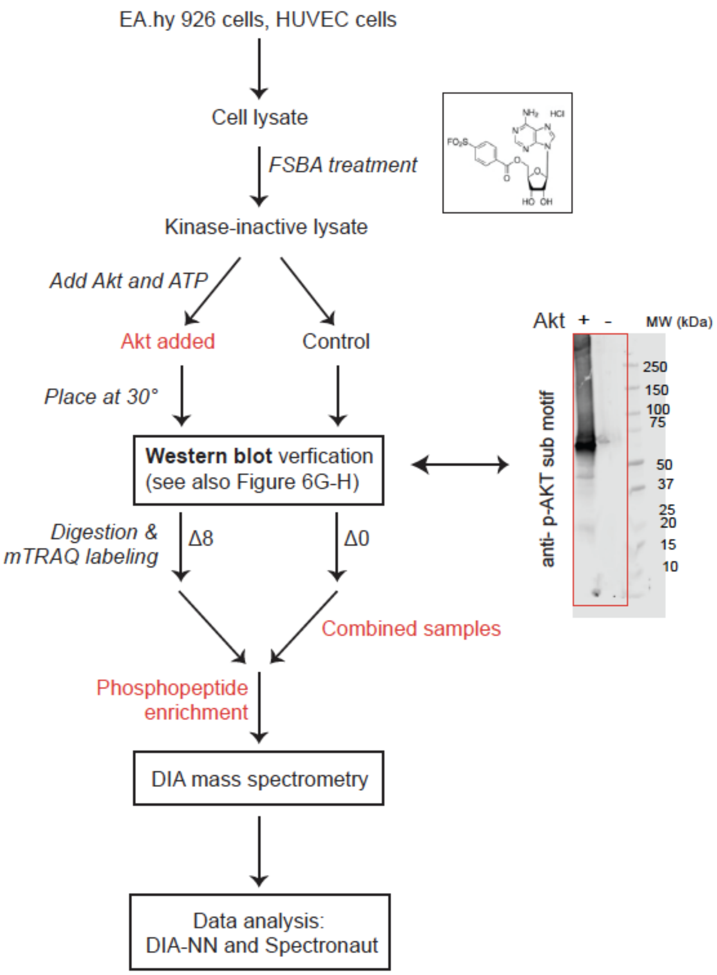

Figure S11. The experimental schema for *in vitro* kinase assay in cell lysate using FSBA and quantitative mass spectrometry. This substrate-finding approach[6] overcomes the obstacle of endogenous protein kinase activity by FSBA (5’-4-fluorosulphonylbenzoyladenosine), an ATP analogue that inhibits protein kinases by occupying the ATP binding site and covalently attaching to an invariant lysine. This approach has several particular advantages over alternative approaches – e.g., it can provide direct substrate identifications in a single experiment. Note, after FSBA treatment, the abundance of state-steady phosphoproteome remained the same, but all the kinases are deactivated by FSBA. Thus, the addition of Akt will not trigger a global downstream phosphoproteome change but only phosphorylation of Akt direct substrates. Herein, we combined the assay with the plexDIA method using mTRAQ labeling [7] before phosphopeptide enrichment. Please see **Methods** for more information.

Supplementary Figure 12

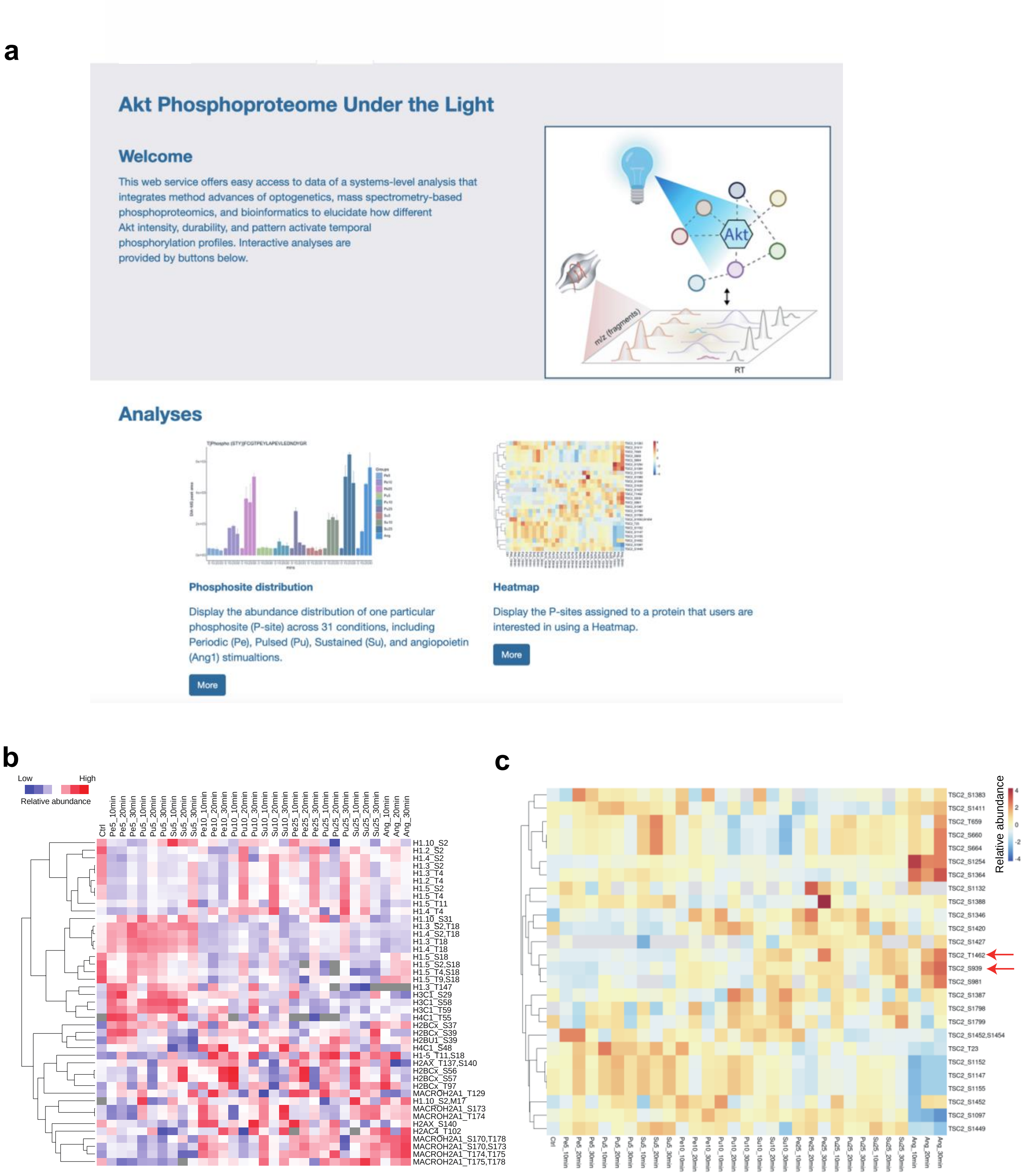

Figure S12. The Akt Optop-DIA Website enabling data navigation and hypothesis generation.
(a) The website hosting data basis for the present study and its interface. **(b)** A total of 43 histone phosphorylation events following different Akt activation conditions were profiled by Optop-DIA. **(c)** A total of 25 unique P-sites were measured for TSC2, including S939, S981, S1132, S1462, S1798, and S1799 carrying Akt motif. Only S939 and S1462 (marked by red arrows) were identified to be potential Akt substrate P-sites in OptoCore.
For more information, please refer to https://yslproteomics.shinyapps.io/AKTPhos/.

References

1. Xu Y, Nan D, Fan J, Bogan JS, Toomre D. Optogenetic activation reveals distinct roles of PIP3 and Akt in adipocyte insulin action. *J Cell Sci* **129**, 2085-2095 (2016).

2. Idevall-Hagren O, Dickson EJ, Hille B, Toomre DK, De Camilli P. Optogenetic control of phosphoinositide metabolism. *Proceedings of the National Academy of Sciences of the United States of America* **109**, E2316-2323 (2012).

3. Zhou Y, *et al.* Metascape provides a biologist-oriented resource for the analysis of systems-level datasets. *Nature communications* **10**, 1523 (2019).

4. Conesa A, Nueda MJ, Ferrer A, Talon M. maSigPro: a method to identify significantly differential expression profiles in time-course microarray experiments. *Bioinformatics* **22**, 1096-1102 (2006).

5. Johnson JL, *et al.* An atlas of substrate specificities for the human serine/threonine kinome. *Nature* **613**, 759-766 (2023).

6. Knight JD, *et al.* A novel whole-cell lysate kinase assay identifies substrates of the p38 MAPK in differentiating myoblasts. *Skelet Muscle* **2**, 5 (2012).

7. Derks J, *et al.* Increasing the throughput of sensitive proteomics by plexDIA. *Nature biotechnology*, (2022).
