# Supplementary material for: An optogenetic-phosphoproteomic study reveals dynamic Akt1 signaling profiles in endothelial cells
Source: Nat Commun. 2023 Jun 26;14:3803. doi: 10.1038/s41467-023-39514-1 (PMC10293293; doi:10.1038/s41467-023-39514-1)
Supplement: Supplementary file 3 — Reporting Summary [file 41467_2023_39514_MOESM3_ESM.pdf]

nature portfolio

Corresponding author(s): William C. Sessa, Yansheng Liu

Last updated by author(s): May 22, 2023

Reporting Summary

Nature Portfolio wishes to improve the reproducibility of the work that we publish. This form provides structure for consistency and transparency in reporting. For further information on Nature Portfolio policies, see our Editorial Policies and the Editorial Policy Checklist.

Statistics

For all statistical analyses, confirm that the following items are present in the figure legend, table legend, main text, or Methods section.

n/a	Confirmed	
☐	☒	The exact sample size (*n*) for each experimental group/condition, given as a discrete number and unit of measurement
☐	☒	A statement on whether measurements were taken from distinct samples or whether the same sample was measured repeatedly
☐	☒	The statistical test(s) used AND whether they are one- or two-sided<br>*Only common tests should be described solely by name; describe more complex techniques in the Methods section.*
☒	☐	A description of all covariates tested
☒	☐	A description of any assumptions or corrections, such as tests of normality and adjustment for multiple comparisons
☐	☒	A full description of the statistical parameters including central tendency (e.g. means) or other basic estimates (e.g. regression coefficient) AND variation (e.g. standard deviation) or associated estimates of uncertainty (e.g. confidence intervals)
☐	☒	For null hypothesis testing, the test statistic (e.g. *F*, *t*, *r*) with confidence intervals, effect sizes, degrees of freedom and *P* value noted<br>*Give P values as exact values whenever suitable.*
☒	☐	For Bayesian analysis, information on the choice of priors and Markov chain Monte Carlo settings
☒	☐	For hierarchical and complex designs, identification of the appropriate level for tests and full reporting of outcomes
☒	☐	Estimates of effect sizes (e.g. Cohen's *d*, Pearson's *r*), indicating how they were calculated

Our web collection on statistics for biologists contains articles on many of the points above.

Software and code

Policy information about availability of computer code

Data collection	Commercial Software: Thermo Xcalibur 4.2.47.
Data analysis	The data are collected with published softwares with parameters specified in the method.<br>Spectronaut Professional (v15 and v17), DIA-NN (v1.8.1 beta 7)<br>R 4.2.1 (https://www.r-project.org/) and R Studio 2021.09.0-351 or higher (https://www.rstudio.com/products/rstudio/download/); R packages: R Shiny interactive web application (v1.7.3), maSigPro (v1.70.0), pheatmap (v1.0.8), and ggplot2 (v3.4.0).<br>David Bioinformatics (v6.8), Perseus (v1.6.14.0), Cytoscape (v3.9.1), PHONEMeS (v2.0.1), PhosR (v1.8.0), GraphPad (v9.4.0), ImageJ (v1.53q), Image studio (v5.2), Velocity (v6.5.1), Adobe Illustrator (27.3.1).

For manuscripts utilizing custom algorithms or software that are central to the research but not yet described in published literature, software must be made available to editors and reviewers. We strongly encourage code deposition in a community repository (e.g. GitHub). See the Nature Portfolio guidelines for submitting code & software for further information.

Data

Policy information about availability of data

All manuscripts must include a data availability statement. This statement should provide the following information, where applicable:

- Accession codes, unique identifiers, or web links for publicly available datasets
- A description of any restrictions on data availability
- For clinical datasets or third party data, please ensure that the statement adheres to our policy

The mass spectrometry-based Optop-DIA datasets, the validation MS datasets, and processed results in this study have been deposited to the ProteomeXchange Consortium via the PRIDE partner repository 108 under accession code PXD034957. [https://proteomecentral.proteomexchange.org/cgi/GetDataset?ID=PXD034957].
The processed data could be also assessed via Akt1 Optop-DIA Website (https://yslproteomics.shinyapps.io/AKTPhos/)
Other publicly available datasets include:
Phosphosite database (PSPdb) [https://www.phosphosite.org/]
Omnipathdb [https://omnipathdb.org/]

Human research participants

Policy information about studies involving human research participants and Sex and Gender in Research.

Reporting on sex and gender	n/a
Population characteristics	n/a
Recruitment	n/a
Ethics oversight	n/a

Note that full information on the approval of the study protocol must also be provided in the manuscript.

Field-specific reporting

Please select the one below that is the best fit for your research. If you are not sure, read the appropriate sections before making your selection.

☒ Life sciences ☐ Behavioural & social sciences ☐ Ecological, evolutionary & environmental sciences

For a reference copy of the document with all sections, see nature.com/documents/nr-reporting-summary-flat.pdf

Life sciences study design

All studies must disclose on these points even when the disclosure is negative.

Sample size	No sample size calculations were performed. The major experiment studied the phosphoproteomic response of the same optogenetic cell line (EA.hy 926 cells) following the Akt phosphorylation level and patterns, including 3 light intensities × 3 patterns × 3 time points, independently repeated in biological duplicates. When one of three traits (intensity, pattern, and time length) was focused, nine samples per group (n=3×3) were used for comparison. Based on our experience and other studies with similar methodologies, it is difficult to calculate the sample size because the quantitative reproducibility can be variable for different phosphosites. However, to infer the differential responsive patterns, 100s of phosphosites were included in each pattern cluster for the analysis by rigorous statistical analysis. The results were independently verified in two normal (non-transduced) cell lines, EA.hy 926 and HUVECs, using independent mass spectrometric assay and western blots.
Data exclusions	No data were excluded.
Replication	All attempts at replication were successful and are presented. All mass spectrometry phosphoproteomic measurements were carried out with two independent biological replicates. For all WB blots, at least two times of experiments were repeated independently with similar results.
Randomization	All biological samples were randomized for phosphoproteomic procession analysis. The two biological replicates were measured for DIA-MS analysis as two batches so that they are entirely independent (not affected by mass spectrometric sensitivity drift, if there is any).
Blinding	The investigator was not blinded to group allocation during data collection or analysis, due to the requirement to allocate chromatographic cleaning runs during the LC-MS analysis (so that the carry-over peptides can be removed if there is any) and the purpose of identifying co-varying phosphosites with Akt activation patterns and temporal length.

Reporting for specific materials, systems and methods

We require information from authors about some types of materials, experimental systems and methods used in many studies. Here, indicate whether each material, system or method listed is relevant to your study. If you are not sure if a list item applies to your research, read the appropriate section before selecting a response.

Materials & experimental systems

n/a	Involved in the study
☐	☒ Antibodies
☐	☒ Eukaryotic cell lines
☒	☐ Palaeontology and archaeology
☒	☐ Animals and other organisms
☒	☐ Clinical data
☒	☐ Dual use research of concern

Methods

n/a	Involved in the study
☒	☐ ChIP-seq
☒	☐ Flow cytometry
☒	☐ MRI-based neuroimaging

Antibodies

Antibodies used

monoclonal rabbit phospho-Akt T308 (cell signaling, Cat#2965); monoclonal rabbit phospho-Akt S473 (cell signaling, Cat#9271); monoclonal mouse pan-Akt (cell signaling,Cat#2920); chicken polyclonal anti-GFP (Abcam, Cat#ab13970); monoclonal rabbit phospho-eNOS S1177 (cell signaling; Cat#9570); polyclonal rabbit eNOS (cell signaling, Cat#9572); monoclonal rabbit phospho-Erk T202/Tyr204 (cell signaling; Cat#4370); monoclonal rabbit Erk (cell signaling; Cat#4695); polyclonal rabbit phospho-GSK-3β S9 (cell signaling, Cat#9336); monoclonal rabbit GSK (cell signaling, Cat#9315); Polyclonal Rabbit Phospho-NDRG1 Ser330 (cell signaling, Cat#3506); Polyclonal Rabbit NDRG1 (cell signaling, Cat#5196); Polyclonal Rabbit Phospho-NEDD4L Ser448 (cell signaling, Cat#8063); Polyclonal Rabbit NEDD4L Antibody (cell signaling, Cat#4013); monoclonal mouse Hsp90 (BD Biosciences; Cat# 610419); and monoclonal mouse β-Actin (Sigma-Aldrich, Cat# A5441); goat anti-rabbit Alexa Fluor 680 (Thermo Fisher, Cat#A20984); goat anti-chicken Alexa Fluor 488 (Thermo Fisher, Cat#A11039), goat anti-mouse Alexa Fluor 680 (Thermo Fisher, Cat#A21057); goat anti-mouse 800 (Rockland, Cat#610-145-002-0.5).
Please see the antibody dilution information in Supplementary Table 1 (all at 1:1000).

Validation

All antibodies used in this study were obtained from reputable commercial vendors. Any validation statements are available on the manufacturer's website, for example, monoclonal rabbit phospho-Akt T308 (cell signaling, Cat#2965) was validated in-house per statement from manufacturer; chicken polyclonal anti-GFP (Abcam, Cat#ab13970) was validated with knockout edited cell lines for per manufacturer statement.

Cell Signaling Technology: "This product has met all of the quality control standards defined by Cell Signaling Technology, Inc."
Abcam: "Our Abpromise guarantee covers the use of it in the following tested applications."
Thermo Scientific: "Specificity testing is combined with extensive application validation data to provide confidence that our high-quality antibodies will help enable superior performance."
Sigma :"Sigma-Aldrich warrants, that at the time of the quality release or subsequent retest date this product conformed to the information contained in this publication. "

Example of citations for primary antibodies.
phospho-Akt T308 (cell signaling, Cat#2965): Franke, T.F. et al. (1997) Cell 88, 435-7.
phospho-Akt S473 (cell signaling, Cat#9271): Burgering, B.M. and Coffer, P.J. (1995) Nature 376, 599-602.
pan-Akt (cell signaling,Cat#2920): Sarbassov, D.D. et al. (2005) Science 307, 1098-101.
phospho-eNOS S1177 (cell signaling; Cat#9570): Fulton, D. et al. (1999) Nature 399, 597-601.
eNOS (cell signaling, Cat#9572): Bird, A.W. et al. (2002) Nature 419, 411-5.
phospho-Erk T202/Tyr204 (cell signaling; Cat#4370): Marais, R. et al. (1993) Cell 73, 381-93.
Erk (cell signaling; Cat#4695): Marais, R. et al. (1993) Cell 73, 381-93.
phospho-GSK-3β S9 (cell signaling, Cat#9336): Cross, D.A. et al. (1995) Nature 378, 785-9.
GSK (cell signaling, Cat#9315): Nusse, R. (1997) Cell 89, 321-3
Phospho-NDRG1 Ser330 (cell signaling, Cat#3506): Stein, S. et al. (2004) J Biol Chem 279, 48930-40.
NDRG1 (cell signaling, Cat#5196): Stein, S. et al. (2004) J Biol Chem 279, 48930-40.
Phospho-NEDD4L Ser448 (cell signaling, Cat#8063): Snyder, P.M. et al. (2004) J Biol Chem 279, 45753-8.
NEDD4L Antibody (cell signaling, Cat#4013): Goulet, C.C. et al. (1998) J Biol Chem 273, 30012-7.
Hsp90 (BD Biosciences; Cat# 610419): Zou, M., et al. (2017). Oncogene 36(15); 2160-2171
β-Actin (Sigma-Aldrich, Cat# A5441): Mousa, H. et al. Cancer Genomics Proteomics. 16: 505-518.

Finally, in this study, the antibodies are used for the purpose of mass spectrometry data validation, meaning that the conclusion derived from the antibodies are also validated by quantitative mass spectrometry.

Eukaryotic cell lines

Policy information about cell lines and Sex and Gender in Research

Cell line source(s)

EA.hy926 (ATCC, Cat# CRL-2922); HEK 293T (ATCC, Cat# CRL-3216); HeLa (ATCC, Cat# CCL-2); and HUVEC (Yale VBT program, Item number T25)

Authentication	EA.hy926, Hela cells, and HEK293 cells were initially authenticated by ATCC through STR profiling and karyotyping. HUVEC cells were certified by Yale Vascular Biology & Therapeutics Program, for which flow cytometry and immunostaining for endothelial markers such as CD31 and VE-CAD were performed to authenticate HUVEC cells
Mycoplasma contamination	No mycoplasma contamination - testing for mycoplasma performed with PCR-based Venor GeM Mycoplasma Detection Kit(catalog#MP0025, Sigma).
Commonly misidentified lines (See ICLAC register)	No commonly misidentified cell lines were used in this study.
