# Supplementary material for: An optogenetic-phosphoproteomic study reveals dynamic Akt1 signaling profiles in endothelial cells
Source: Nat Commun. 2023 Jun 26;14:3803. doi: 10.1038/s41467-023-39514-1 (PMC10293293; doi:10.1038/s41467-023-39514-1)
Supplement: Supplementary file 4 — Description of Additional Supplementary Files [file 41467_2023_39514_MOESM4_ESM.pdf]

<u>Description Of Additional Supplementary Files Document</u>

File Name: Supplementary Data 1

Description: The data basis for Optop-DIA study on Akt. This phosphopeptide-level table contains the quantitative data and clustering results for the present entire investigation. All the data are identified and filtered as described in the Methods and Results. Column A-G provide the information of Swissprot accessions, Gene names, Protein description, the PTM localization within the proteins, peptide charge, and a short name combining gene and phosphosites. Column H-J provide the matching results of the phosphosite as the Akt substrate motif (R-x-R-x-x-S*/T* with R corresponding to arginine, x to any amino acid, and S*/T* representing a phosphorylated serine or threonine) and the more generic kinase motif R-x-x-S*/T*. Column K-AO represent all the log-2 transformed, quantitative DIA-MS peak areas across all 31 conditions including optogenetic and Ang1 stimulation experiments. Column AP lists the Pearson correlation between the particular P site to Akt pT308. Column AQ-AT provide the P-site level clustering results of Figure 2-5. Column AU-AW provide the in or out information for OptoCore list and PSPdb list, as well as the percentile score in the kinome paper of Johnson et al.
