# Supplementary material for: An optogenetic-phosphoproteomic study reveals dynamic Akt1 signaling profiles in endothelial cells
Source: Nat Commun. 2023 Jun 26;14:3803. doi: 10.1038/s41467-023-39514-1 (PMC10293293; doi:10.1038/s41467-023-39514-1)
Supplement: Supplementary file 6 — Source Data [file 41467_2023_39514_MOESM6_ESM.zip › Source Data 2.pdf]

Figure 1b

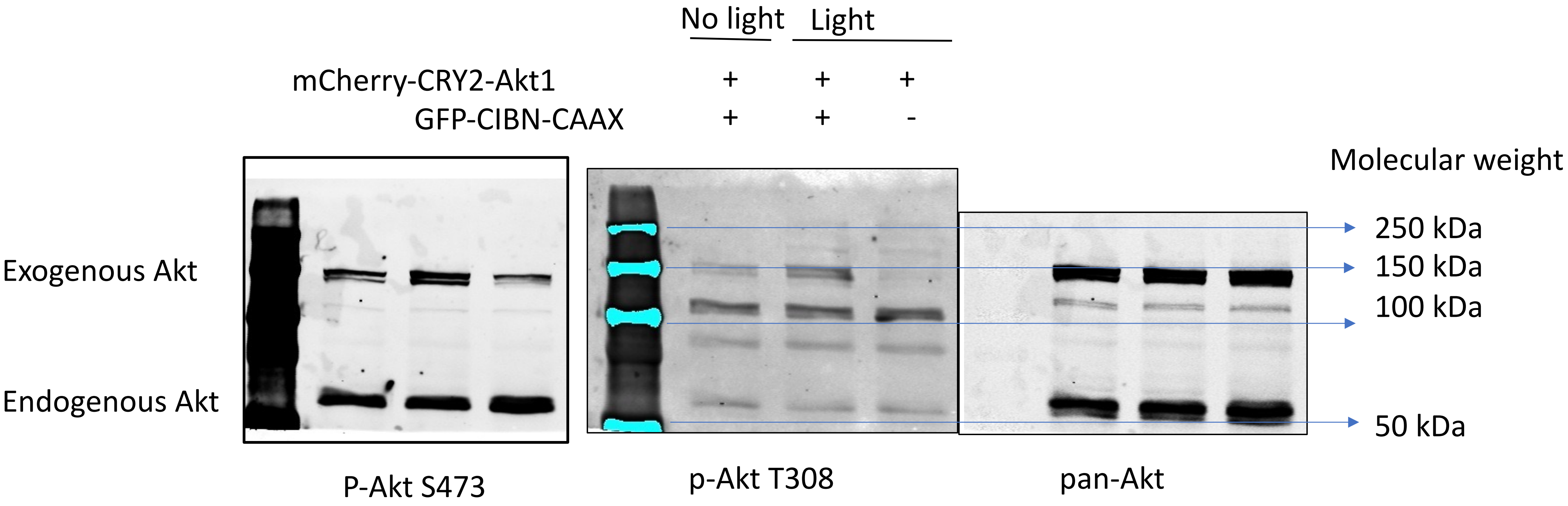

monoclonal rabbit phospho-Akt S473 (Cat#9271, Cell Signaling Technology, at 1:1,000 ratio)

Secondary Antibody: Goat anti-Rabbit Alexa Fluor 680 nm (Thermo Fisher Scientific)

monoclonal rabbit phospho-Akt T308 (Cat#2965, Cell Signaling Technology, at 1:1,000 ratio)

Secondary Antibody: Goat anti-Rabbit Alexa Fluor 680 nm (Thermo Fisher Scientific)

monoclonal mouse pan-Akt (Cat#2920, Cell Signaling Technology, at 1:1,000 ratio)

goat anti-mouse Alexa Fluor 800 (Thermo Fisher Scientific)

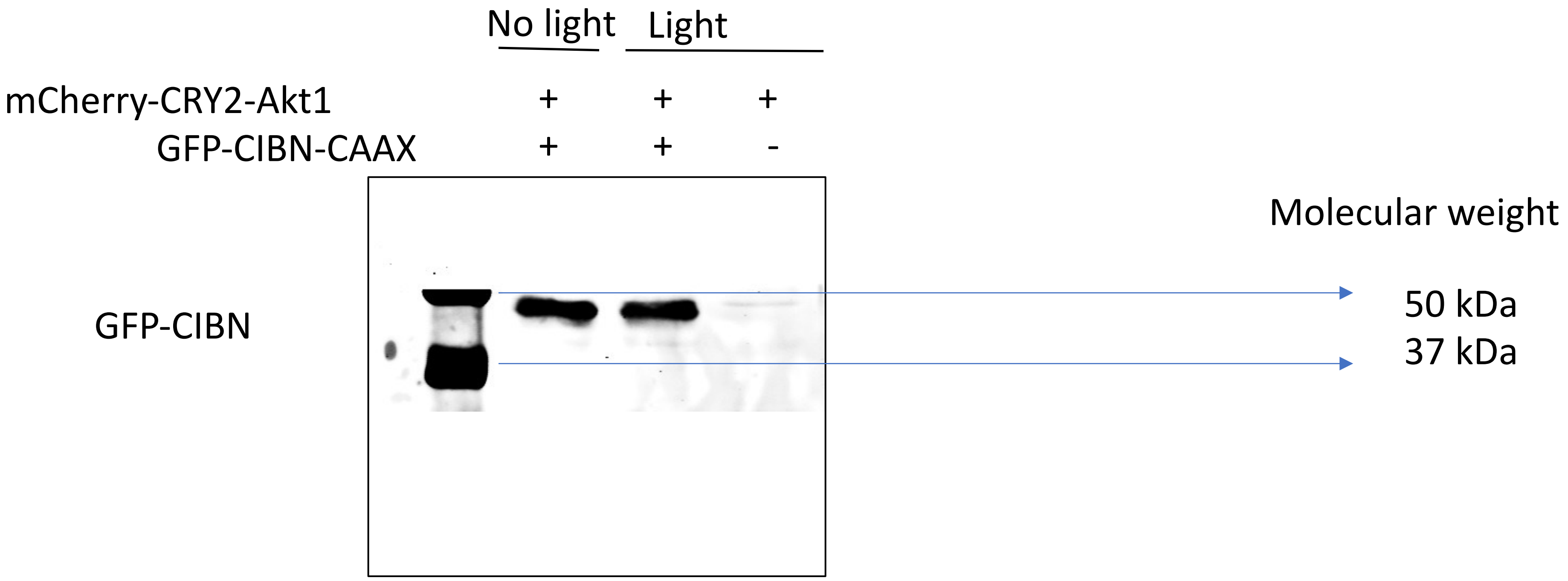

chicken polyclonal anti-GFP (Cat#ab13970, Abcam, at 1:1,000 ratio)

Secondary Antibody: Goat anti-chicken Alexa-Fluor 488 nm (Thermo Fisher Scientific)

Figure 1d

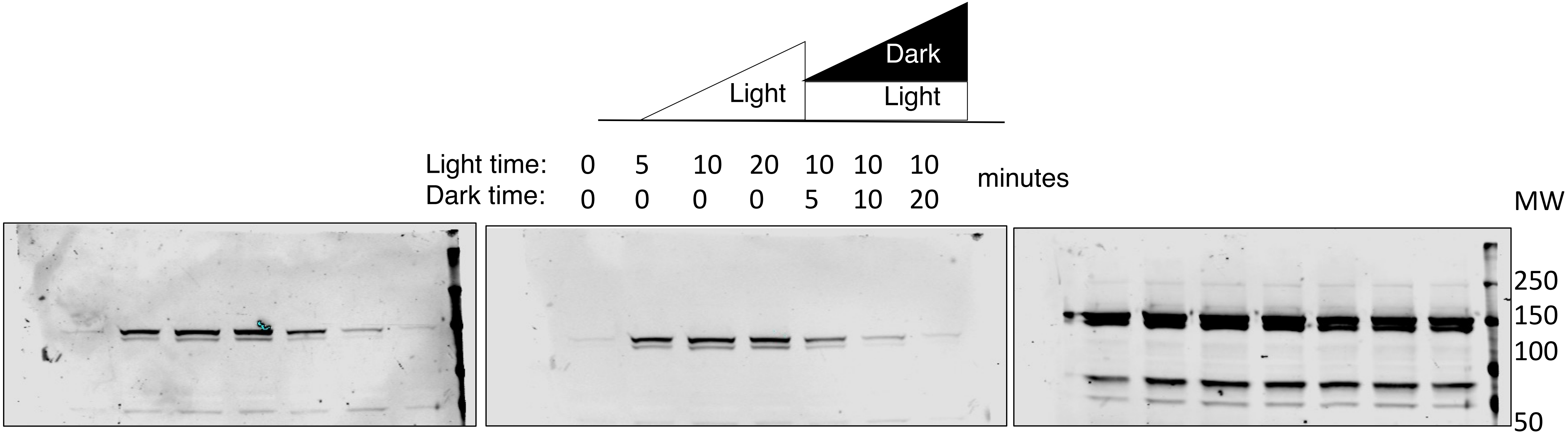

p-T308 Akt

p-S473 Akt

pan-Akt

monoclonal rabbit phospho-Akt S473 (Cat#9271, Cell Signaling Technology, at 1:1,000 ratio)

Secondary Antibody: Goat anti-Rabbit Alexa-Fluor 680 nm (Thermo Fisher Scientific)

monoclonal rabbit phospho-Akt T308 (Cat#2965, Cell Signaling Technology, at 1:1,000 ratio)

Secondary Antibody: Goat anti-Rabbit Alexa-Fluor 800 nm (Thermo Fisher Scientific)

monoclonal mouse pan-Akt (Cat#2920, Cell Signaling Technology, at 1:1,000 ratio)

goat anti-mouse Alexa Fluor 680 (Thermo Fisher Scientific)

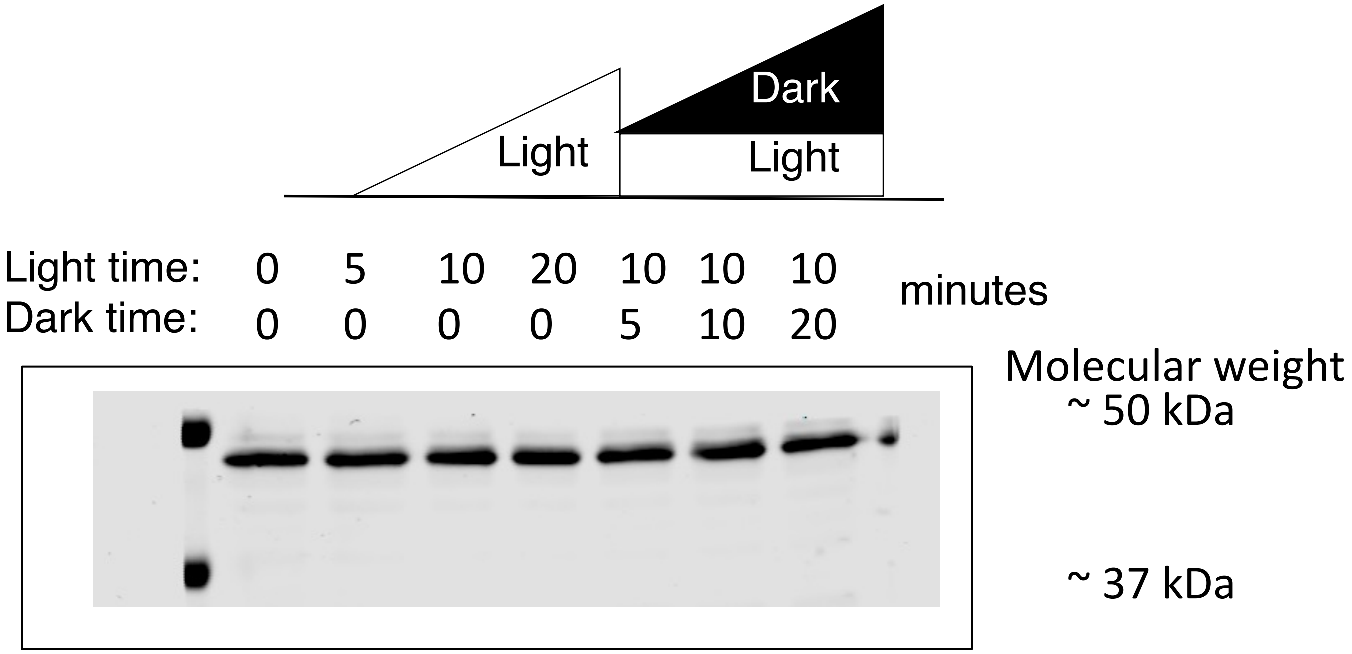

CIBN-GFP-CAAX

chicken polyclonal anti-GFP (Cat#ab13970, Abcam, at 1:1,000 ratio)

Secondary Antibody: Goat anti-chicken Alexa-Fluor 488 nm (Thermo Fisher Scientific)

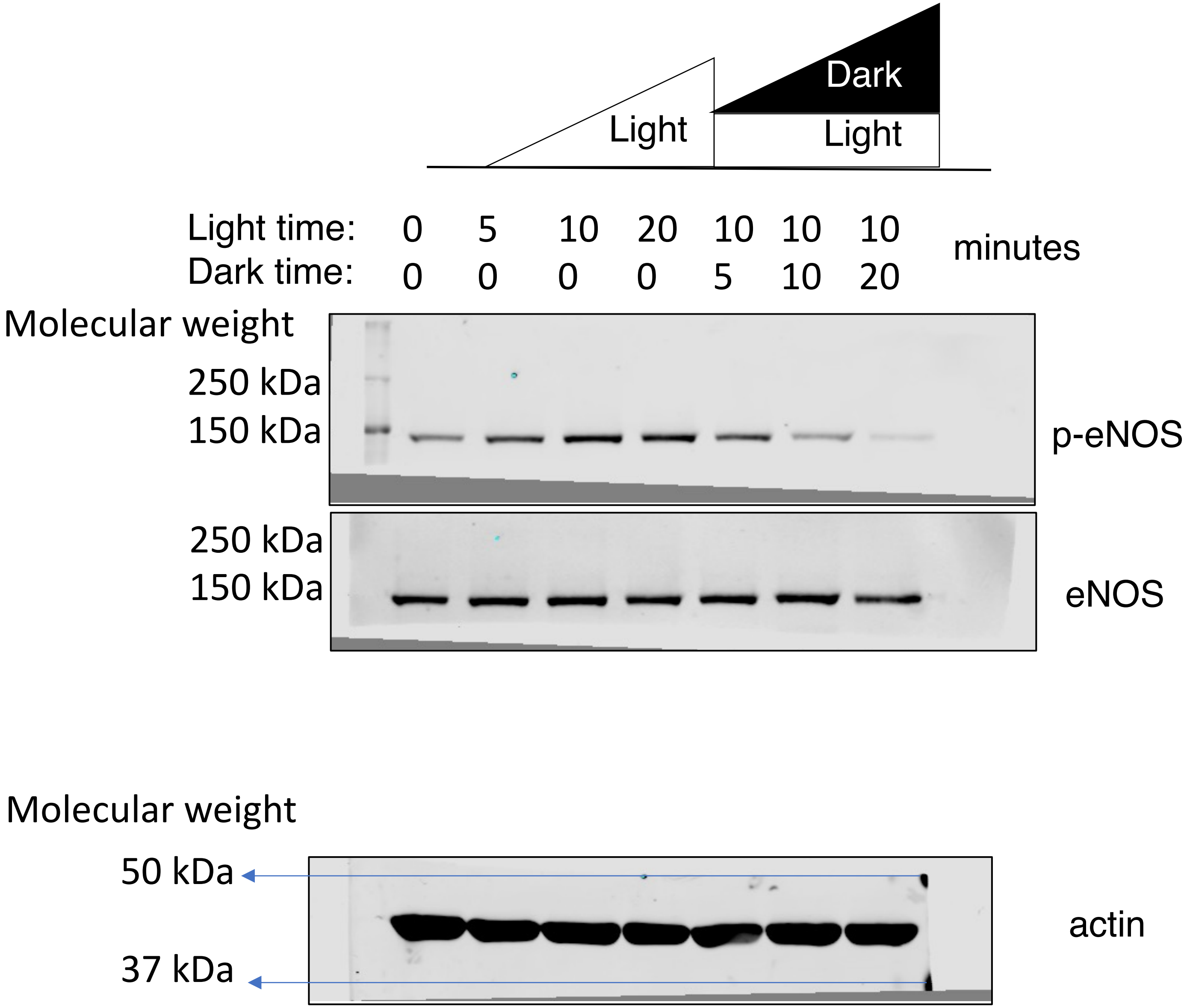

monoclonal rabbit phospho-eNOS S1177 (Cat#9570, Cell Signaling Technology, at 1:1,000 ratio)

Secondary Antibody: Goat anti-Rabbit Alexa-Fluor 680 nm (Thermo Fisher Scientific)

Polyclonal rabbit eNOS (Cat#9572, Cell Signaling Technology, at 1:1,000 ratio)

Secondary Antibody: Goat anti-Rabbit Alexa-Fluor 800 nm (Thermo Fisher Scientific)

monoclonal mouse β-Actin (Cat#A5441, Sigma-Aldrich, at 1:1,000 ratio)

Secondary Antibody: goat anti-mouse Alexa Fluor 680 (Thermo Fisher Scientific)

Figure 1e

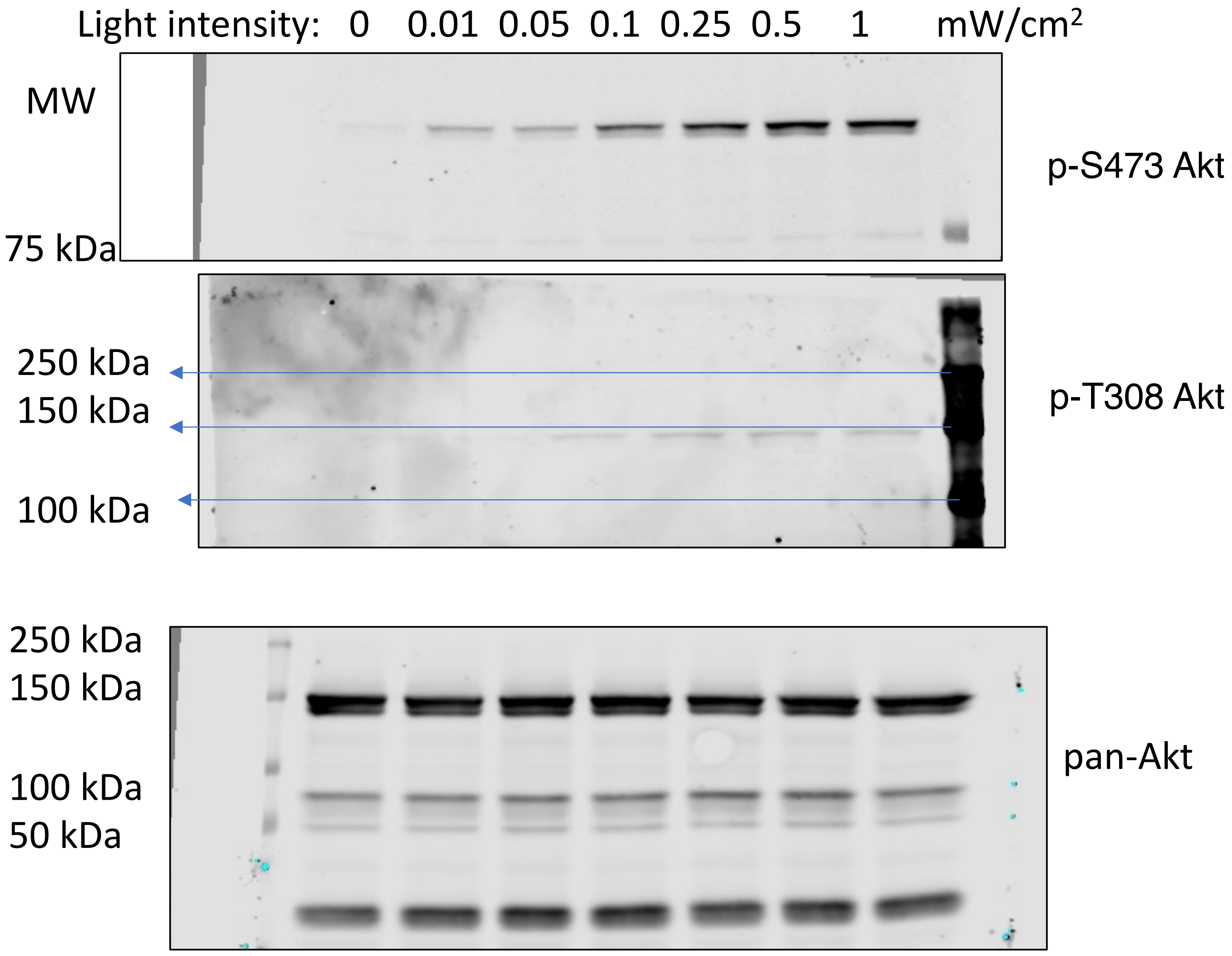

monoclonal rabbit phospho-Akt S473 (Cat#9271, Cell Signaling Technology, at 1:1,000 ratio)

Secondary Antibody: Goat anti-Rabbit Alexa-Fluor 800 nm (Thermo Fisher Scientific)

monoclonal rabbit phospho-Akt T308 (Cat#2965, Cell Signaling Technology, at 1:1,000 ratio)

Secondary Antibody: Goat anti-Rabbit Alexa-Fluor 680 nm (Thermo Fisher Scientific)

monoclonal mouse pan-Akt (Cat#2920, Cell Signaling Technology, at 1:1,000 ratio)

goat anti-mouse Alexa Fluor 680 (Thermo Fisher Scientific)

Light intensity: 0 0.01 0.05 0.1 0.25 0.5 1 mW/cm^2

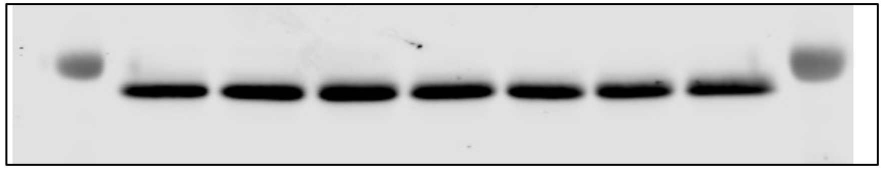

Molecular weight ~ 50 kDa

CIBN-GFP-CAAX

chicken polyclonal anti-GFP (Cat#ab13970, Abcam, at 1:1,000 ratio)

Secondary Antibody: Goat anti-chicken Alexa-Fluor 488 nm (Thermo Fisher Scientific)

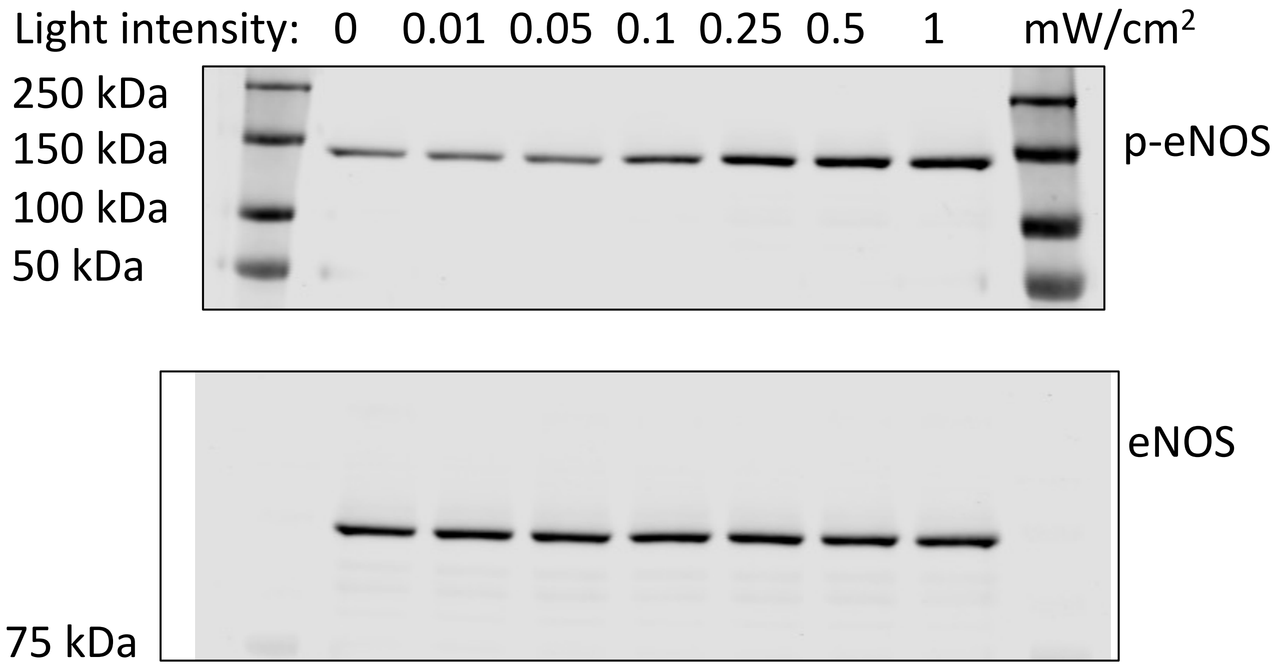

monoclonal rabbit phospho-eNOS S1177 (Cat#9570, Cell Signaling Technology, at 1:1,000 ratio)

Secondary Antibody: Goat anti-Rabbit Alexa-Fluor 680 nm (Thermo Fisher Scientific)

Polyclonal rabbit eNOS (Cat#9572, Cell Signaling Technology, at 1:1,000 ratio)

Secondary Antibody: Goat anti-Rabbit Alexa-Fluor 800 nm (Thermo Fisher Scientific)

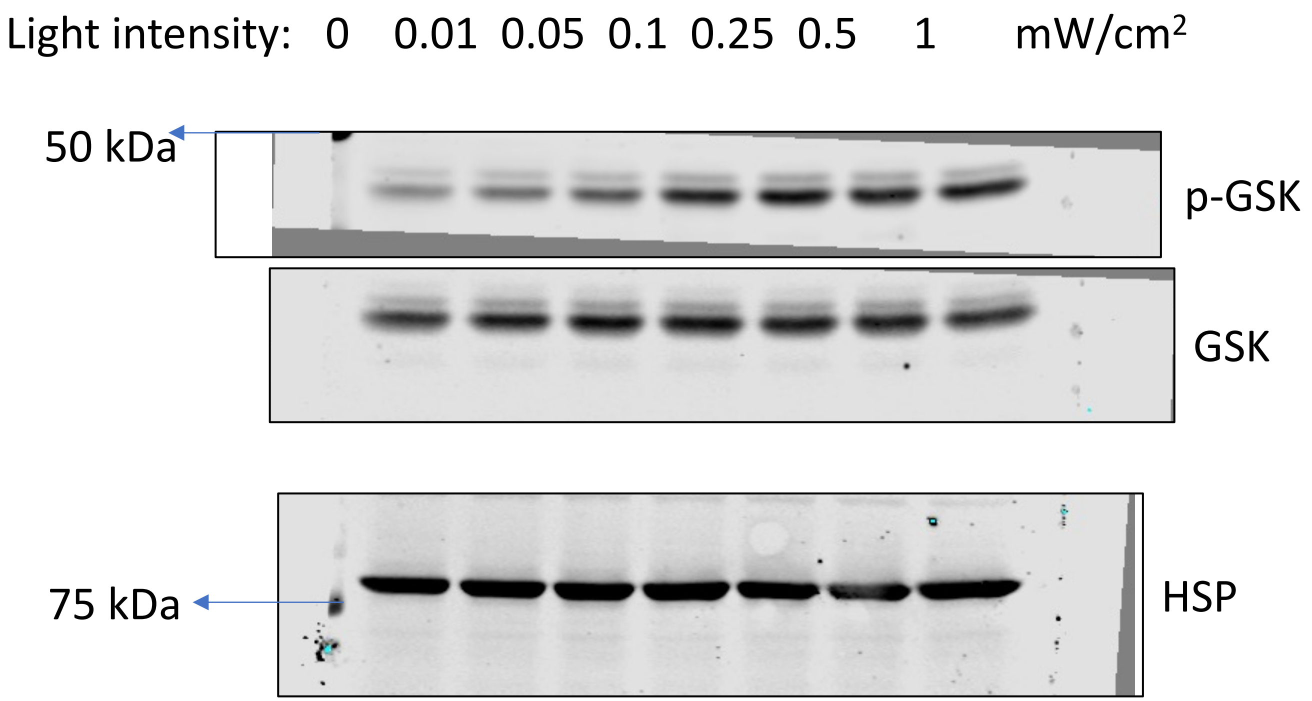

polyclonal rabbit phosphor-GSK-3β S9 (Cat#9336, Cell Signaling Technology, at 1:1,000 ratio)

Secondary Antibody: Goat anti-Rabbit Alexa-Fluor 680 nm (Thermo Fisher Scientific)

polyclonal rabbit phosphor-GSK-3β S9 (Cat#9315, Cell Signaling Technology, at 1:1,000 ratio)

Secondary Antibody: Goat anti-Rabbit Alexa-Fluor 800 nm (Thermo Fisher Scientific)

monoclonal mouse Hsp90 (Cat#610419, BD Biosciences, at 1:1,000 ratio)

Secondary Antibody: Goat anti-Mouse Alexa-Fluor 800 nm (Thermo Fisher Scientific)

Figure 6g

p-eNOS uncropped blot

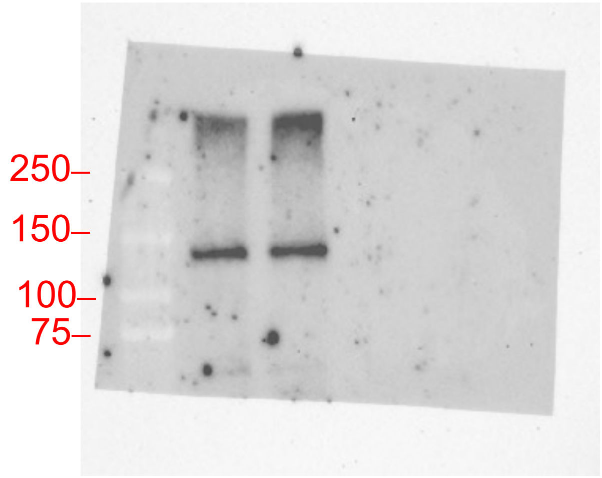

Total eNOS uncropped blot

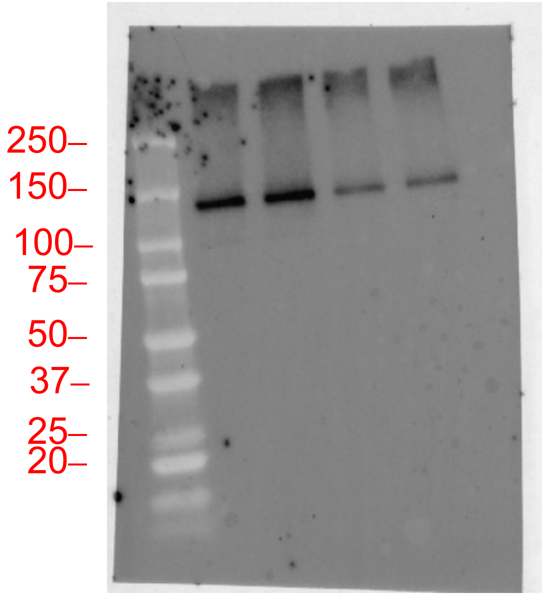

p-GSK3 uncropped blot

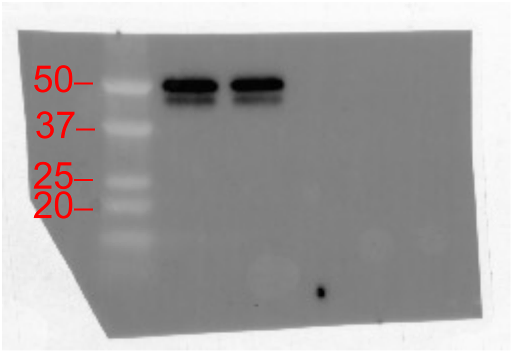

Total GSK3 uncropped blot

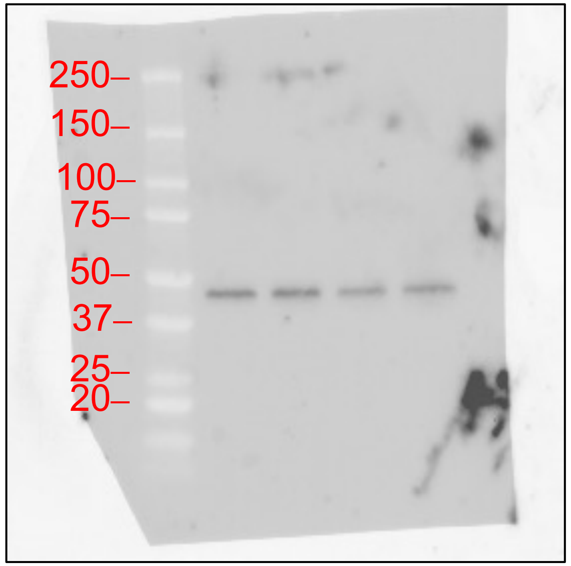

GAPDH uncropped blot

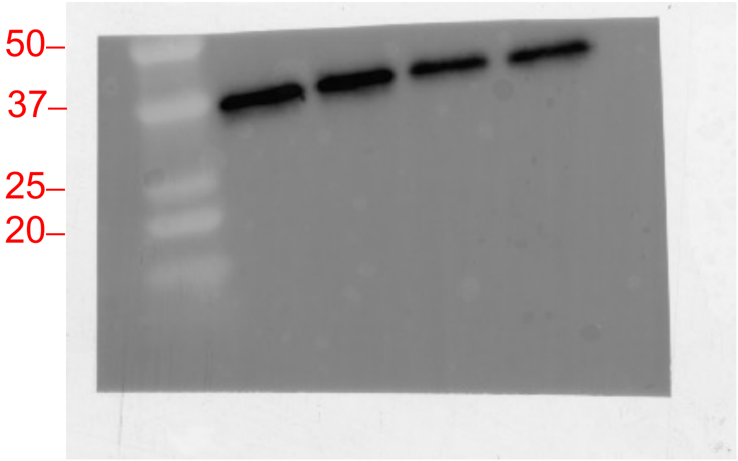

GAPDH uncropped blot

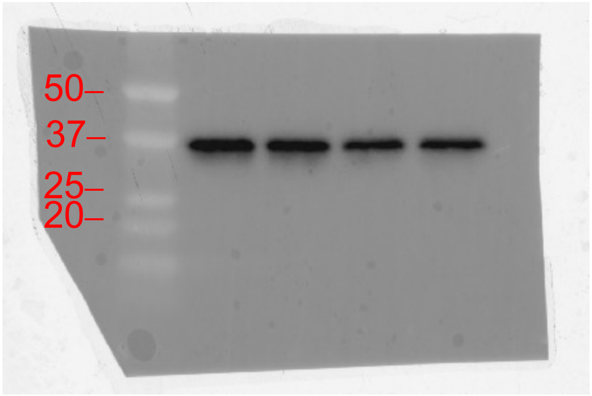

p-NEDD4L uncropped blot

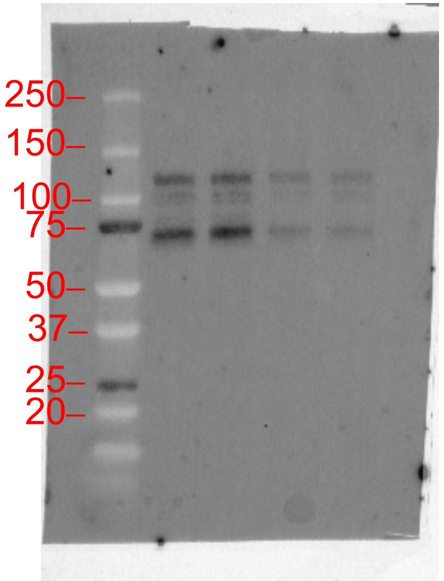

Total NEDD4L uncropped blot

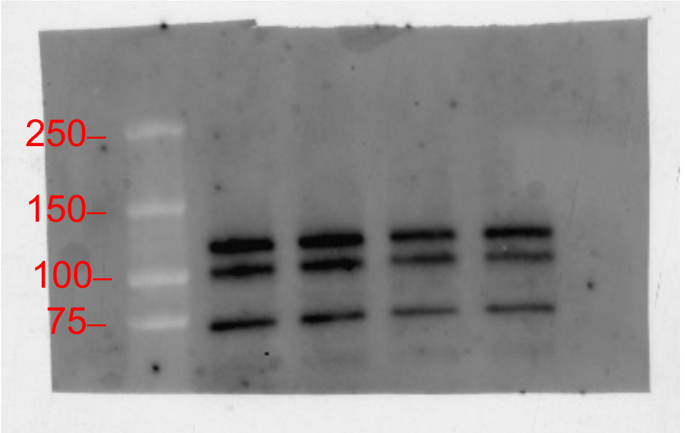

p-NDRG1 uncropped blot

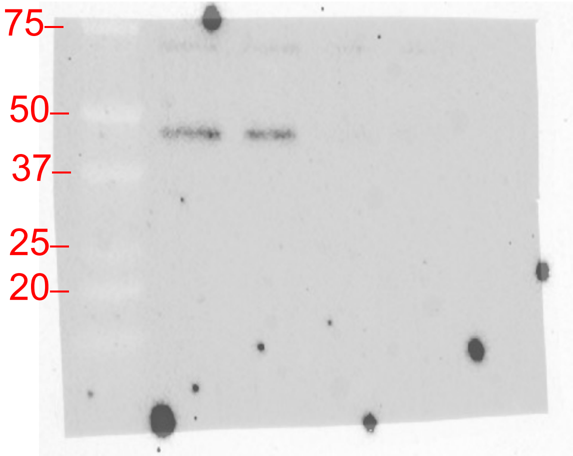

Total NDRG1 uncropped blot

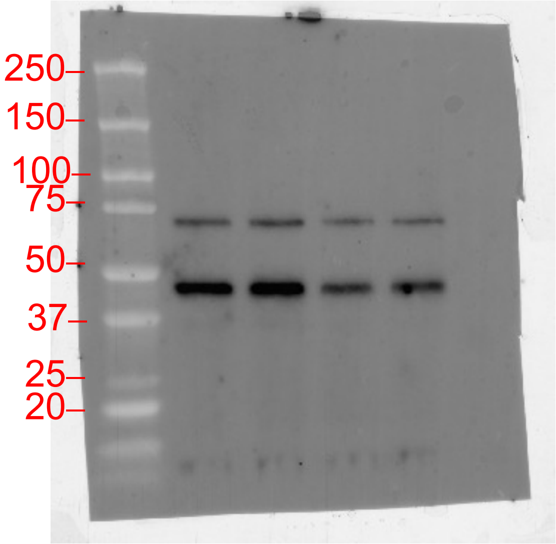

GAPDH uncropped blot

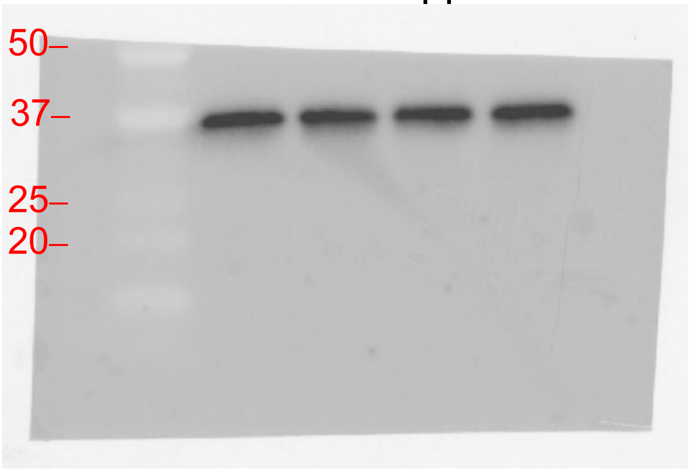

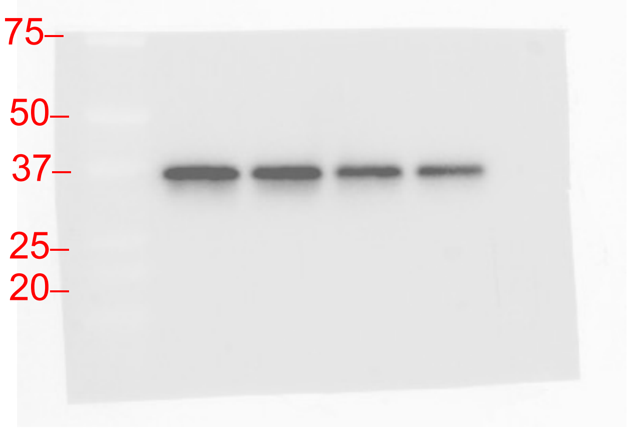

Figure 6h

p-GSK3 uncropped blot

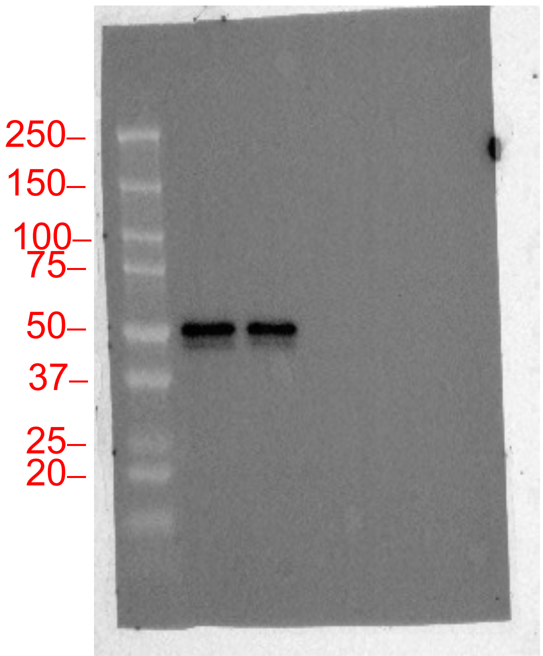

Total GSK3 uncropped blot

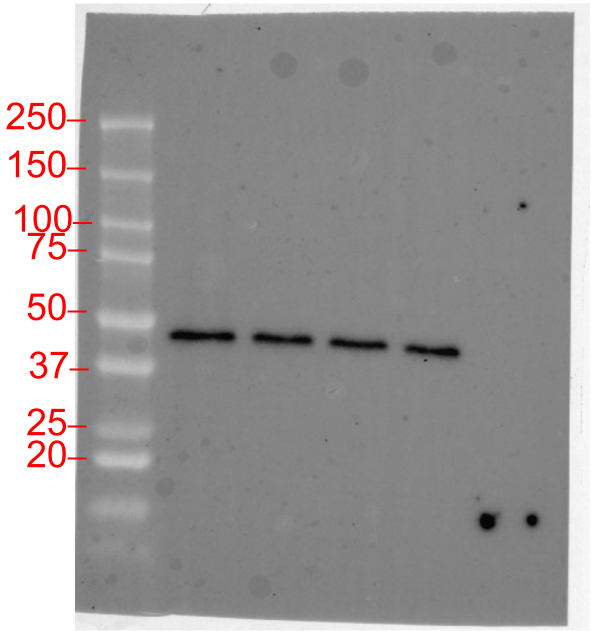

p-eNOS uncropped blot

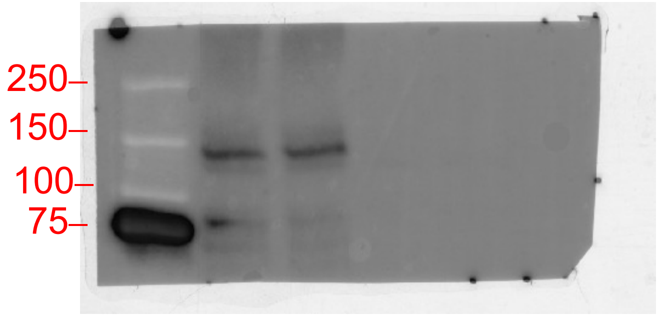

Total eNOS uncropped blot

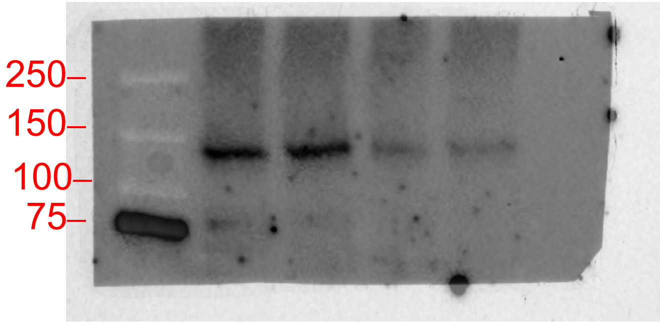

GAPDH uncropped blot

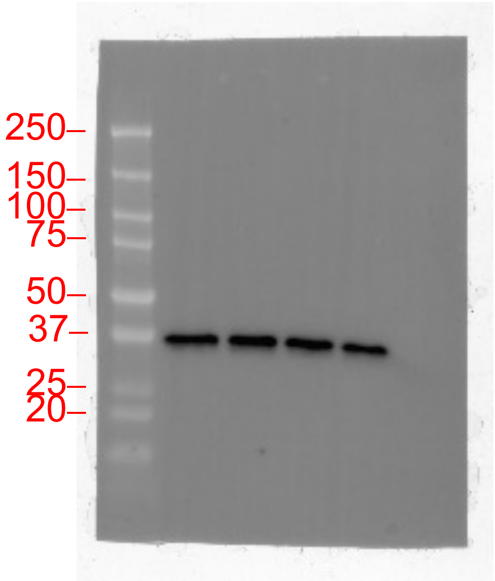

GAPDH uncropped blot

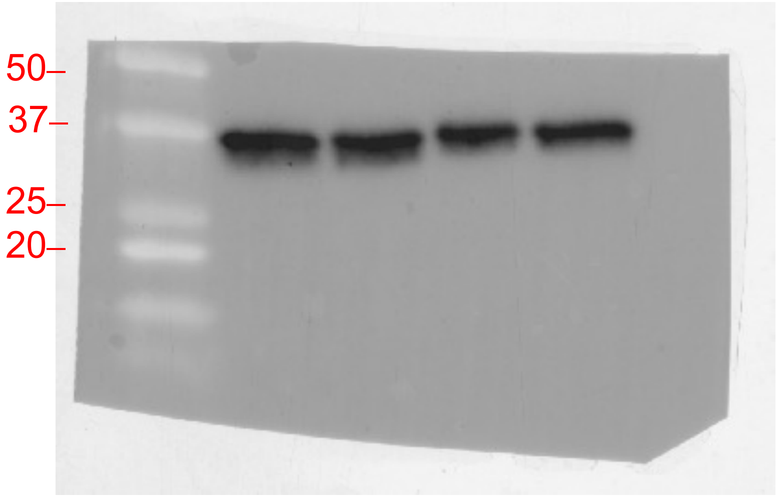

p-NEDD4L uncropped blot

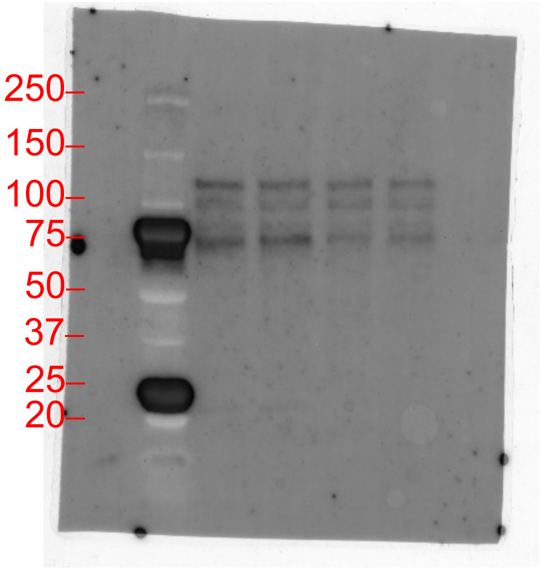

Total NEDD4L uncropped blot

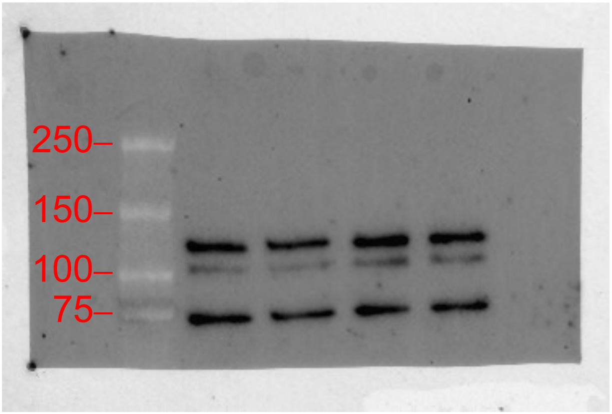

p-NDRG1 uncropped blot

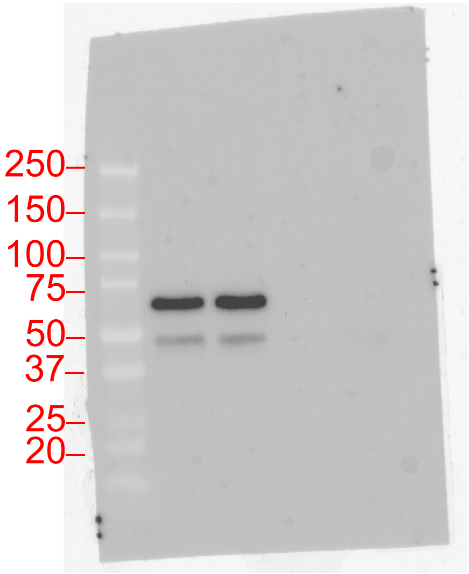

Total NDRG1 uncropped blot

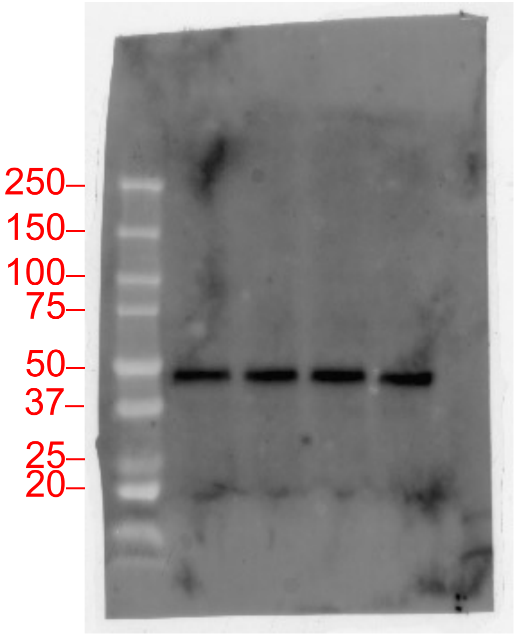

GAPDH uncropped blot

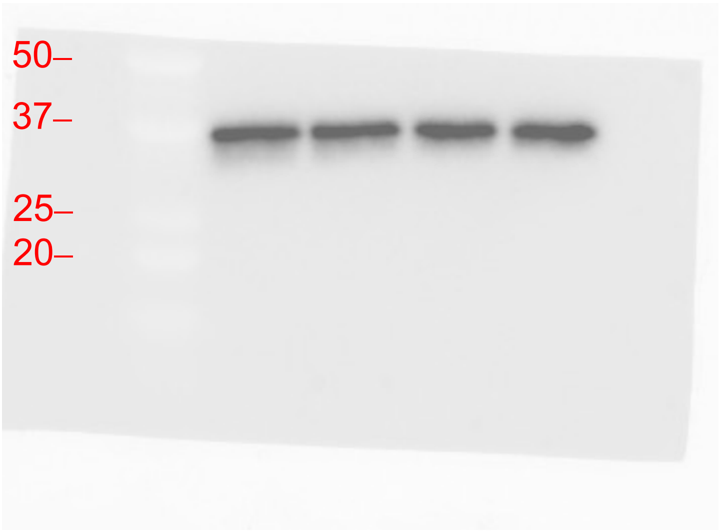

GAPDH uncropped blot

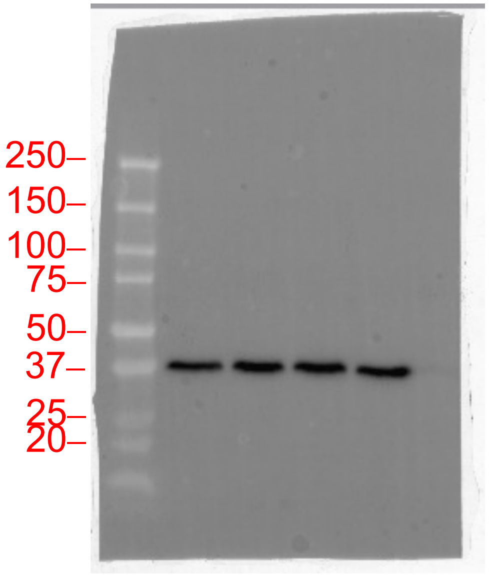

Supplementary Figures

Supplementary Figure 1b

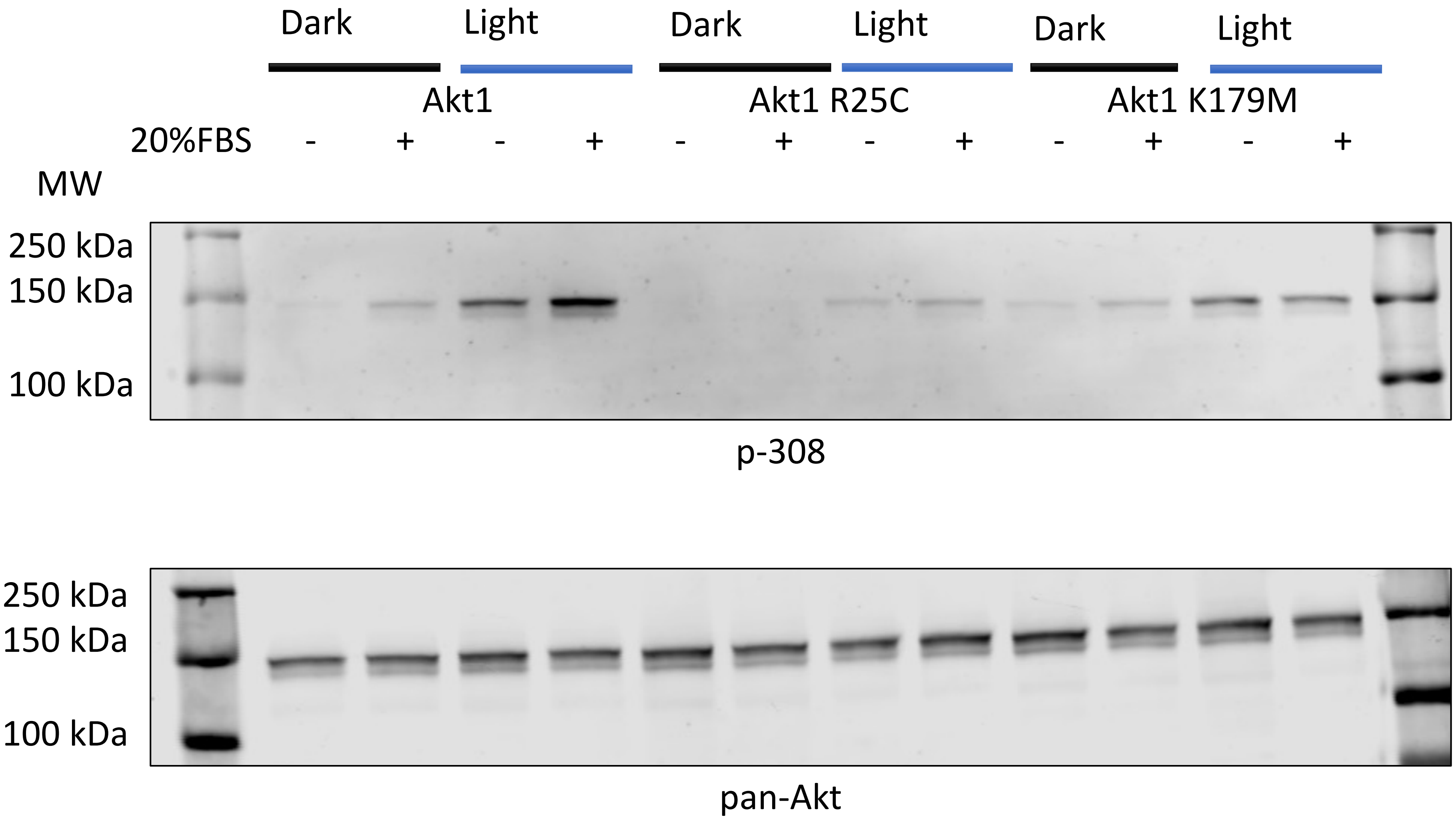

monoclonal rabbit phospho-Akt T308 (Cat#2965, Cell Signaling Technology, at 1:1,000 ratio)

Secondary Antibody: Goat anti-Rabbit Alexa-Fluor 680 nm (Thermo Fisher Scientific)

monoclonal mouse pan-Akt (Cat#2920, Cell Signaling Technology, at 1:1,000 ratio)

goat anti-mouse Alexa Fluor 680 (Thermo Fisher Scientific)

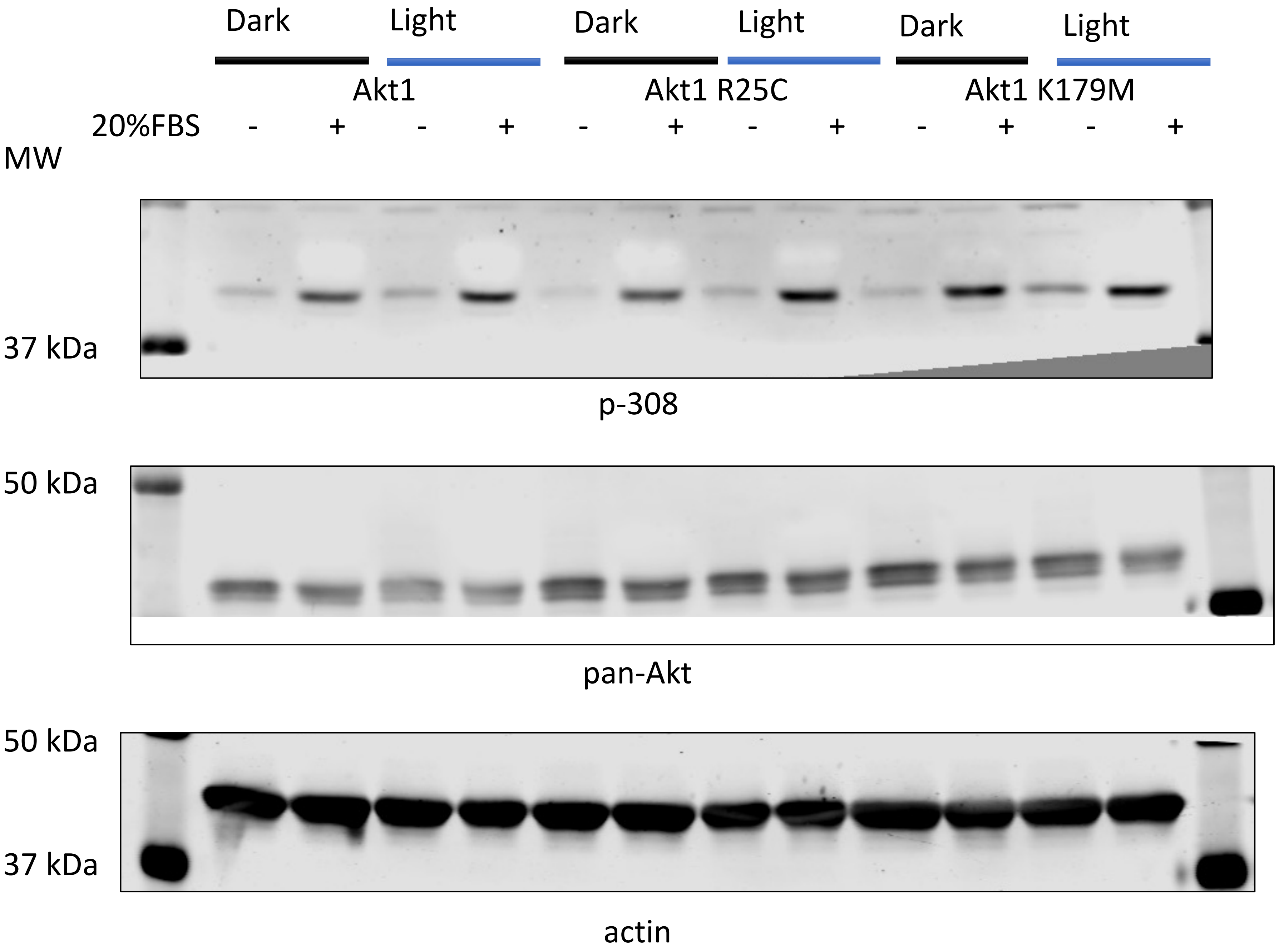

monoclonal rabbit phospho-Akt T308 (Cat#2965, Cell Signaling Technology, at 1:1,000 ratio)

Secondary Antibody: Goat anti-Rabbit Alexa-Fluor 680 nm (Thermo Fisher Scientific)

monoclonal mouse pan-Akt (Cat#2920, Cell Signaling Technology, at 1:1,000 ratio)

goat anti-mouse Alexa Fluor 680 (Thermo Fisher Scientific)

monoclonal mouse β-Actin (Cat#A5441, Sigma-Aldrich, at 1:1,000 ratio)

Secondary Antibody: goat anti-mouse Alexa Fluor 680 (Thermo Fisher Scientific)

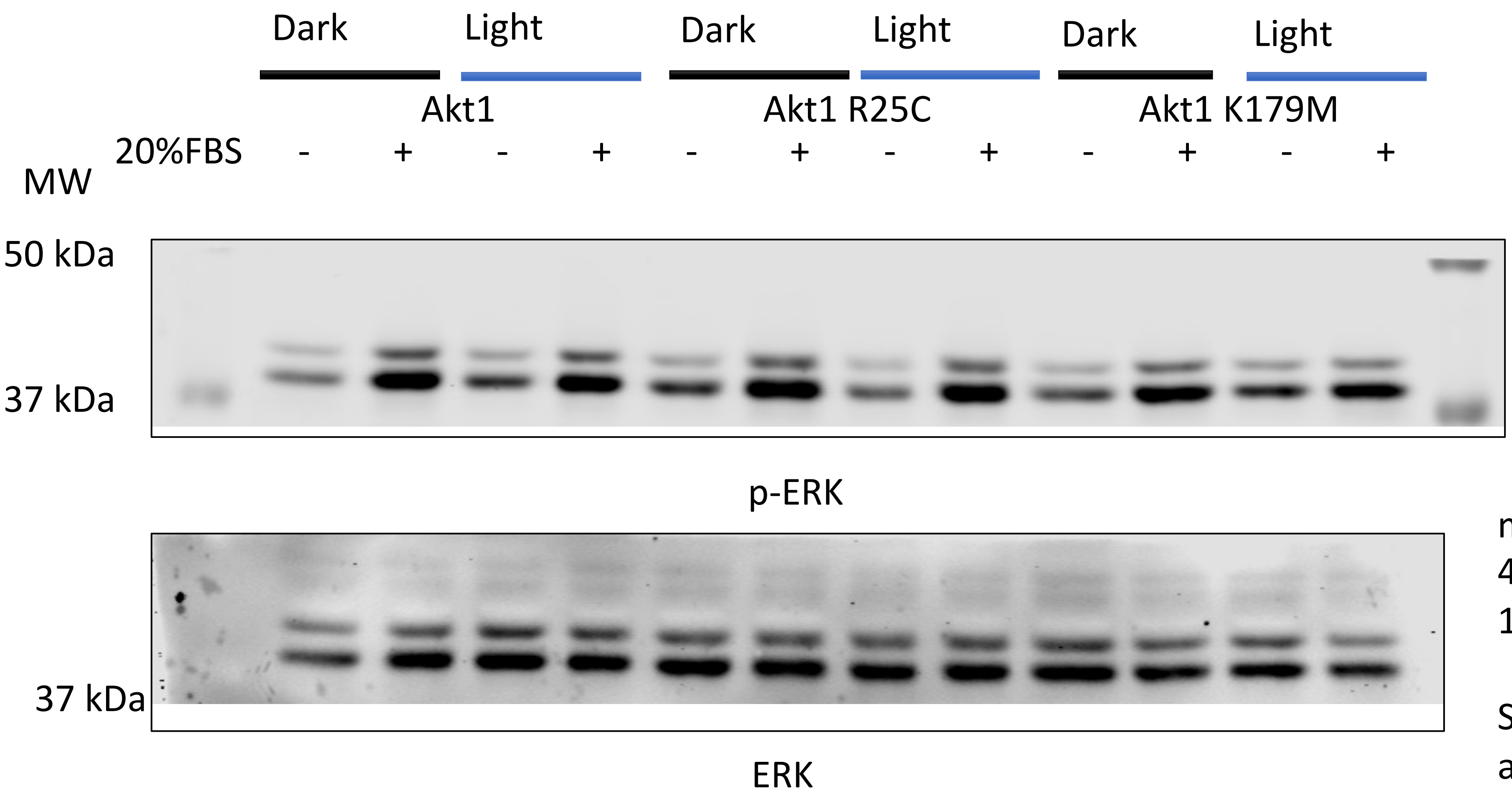

monoclonal rabbit phospho-Erk T202/Tyr204 (Cat#4370 , cell signaling, at 1:1,000 ratio)

Secondary Antibody: Goat anti-rabbit Alexa-Fluor 680 nm (Thermo Fisher Scientific)

monoclonal rabbit Erk (Cat# 4695, cell signaling , at 1:1,000 ratio)

Secondary Antibody: Goat anti-rabbit Alexa-Fluor 800 nm (Thermo Fisher Scientific)

Supplementary Figure 2a

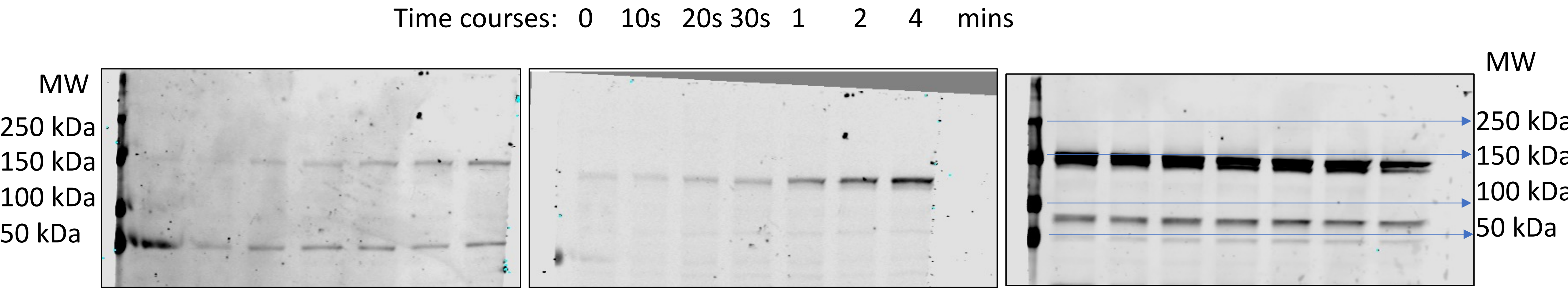

p-T308

monoclonal rabbit phospho-Akt S473 (Cat#9271, Cell Signaling Technology, at 1:1,000 ratio)

Secondary Antibody: Goat anti-Rabbit Alexa Fluor 680 nm (Thermo Fisher Scientific)

p-S473

monoclonal rabbit phospho-Akt T308 (Cat#2965, Cell Signaling Technology, at 1:1,000 ratio)

Secondary Antibody: Goat anti-Rabbit Alexa Fluor 800 nm (Thermo Fisher Scientific)

pan-Akt

monoclonal mouse pan-Akt (Cat#2920, Cell Signaling Technology, at 1:1,000 ratio)

goat anti-mouse Alexa Fluor 680 (Thermo Fisher Scientific)

Time courses: 0 10s 20s 30s 1 2 4 mins

Molecular weight

50 kDa

37 kDa

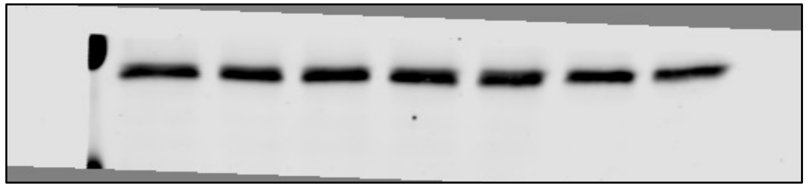

GFP

chicken polyclonal anti-GFP (Cat#ab13970, Abcam, at 1:1,000 ratio)

Secondary Antibody: Goat anti-chicken Alexa-Fluor 488 nm (Thermo Fisher Scientific)

250 kDa

150 kDa

100 kDa

50 kDa

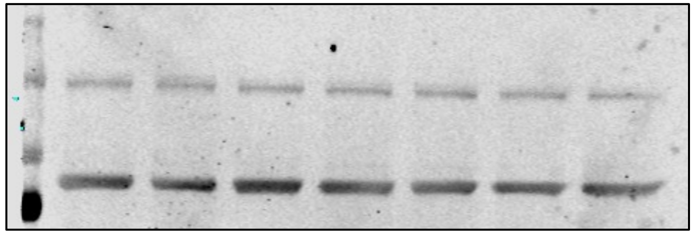

HSP90

monoclonal mouse Hsp90 (Cat#610419, BD Biosciences, at 1:1,000 ratio)

Secondary Antibody: Goat anti-Mouse Alexa-Fluor 800 nm (Thermo Fisher Scientific)

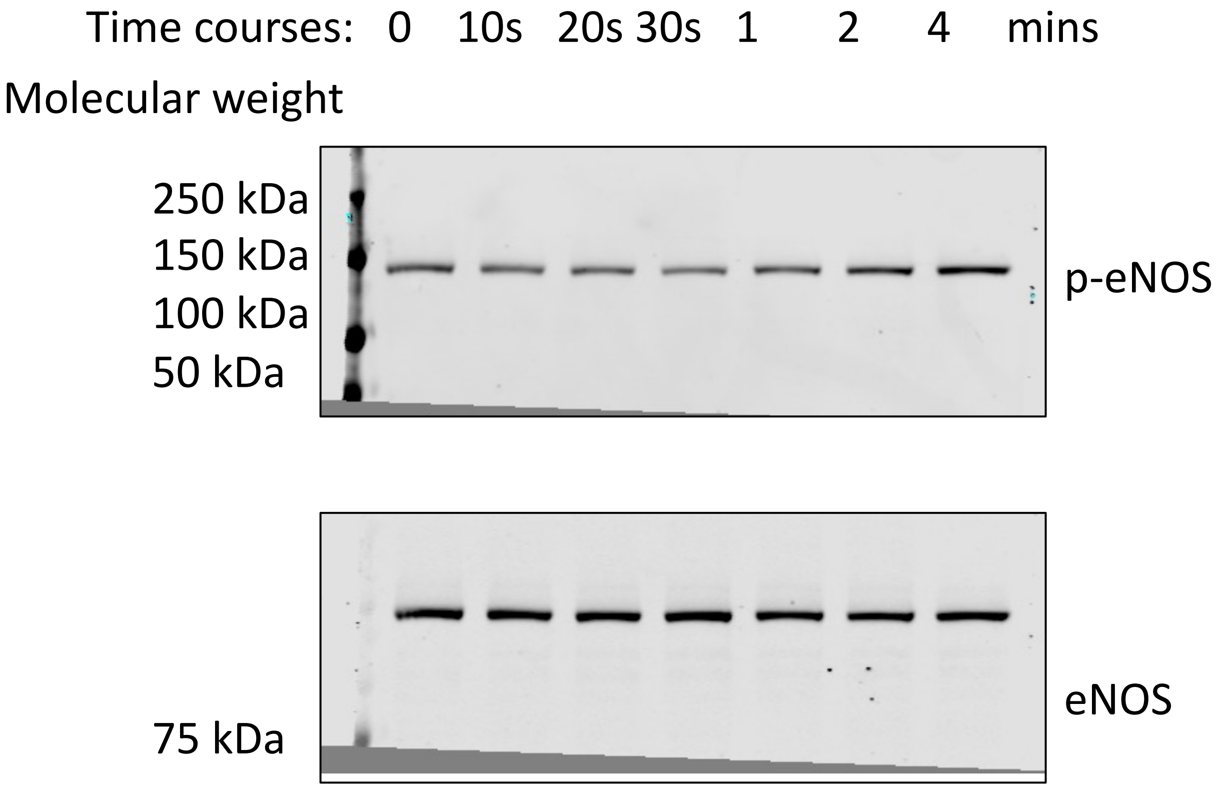

monoclonal rabbit phospho-eNOS S1177 (Cat#9570, Cell Signaling Technology, at 1:1,000 ratio)

Secondary Antibody: Goat anti-Rabbit Alexa-Fluor 680 nm (Thermo Fisher Scientific)

Polyclonal rabbit eNOS (Cat#9572, Cell Signaling Technology, at 1:1,000 ratio)

Secondary Antibody: Goat anti-Rabbit Alexa-Fluor 800 nm (Thermo Fisher Scientific)

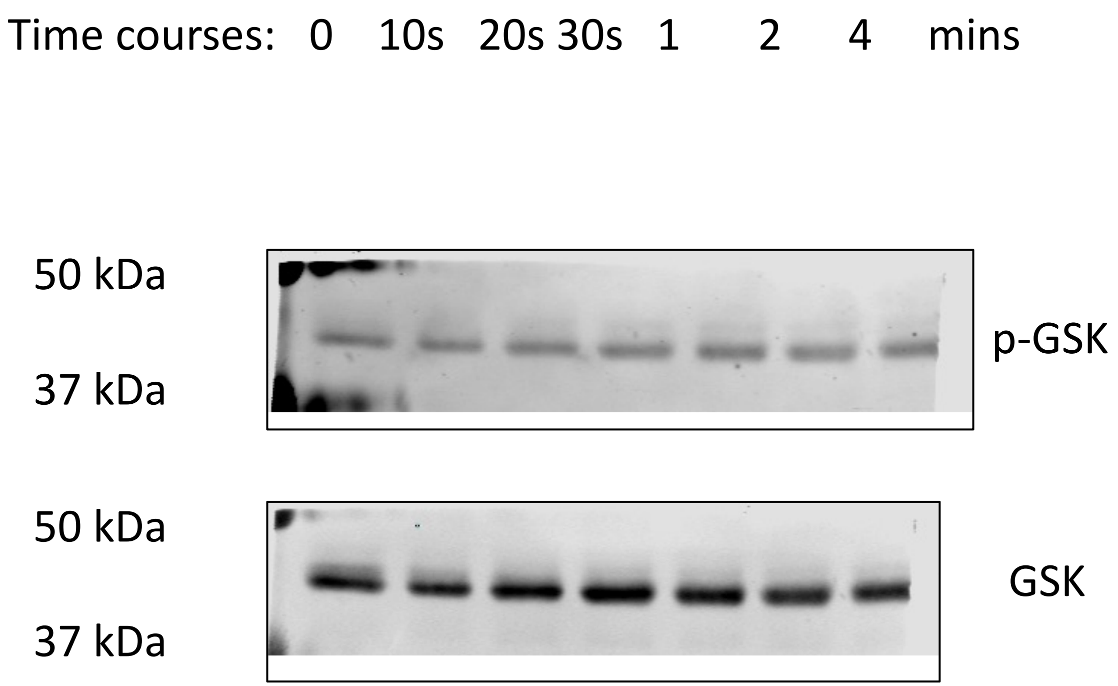

polyclonal rabbit phosphor-GSK-3β S9 (Cat#9336, Cell Signaling Technology, at 1:1,000 ratio)

Secondary Antibody: Goat anti-Rabbit Alexa-Fluor 680 nm (Thermo Fisher Scientific)

polyclonal rabbit phosphor-GSK-3β S9 (Cat#9315, Cell Signaling Technology, at 1:1,000 ratio)

Secondary Antibody: Goat anti-Rabbit Alexa-Fluor 680 nm (Thermo Fisher Scientific)

Supplementary Figure 2b

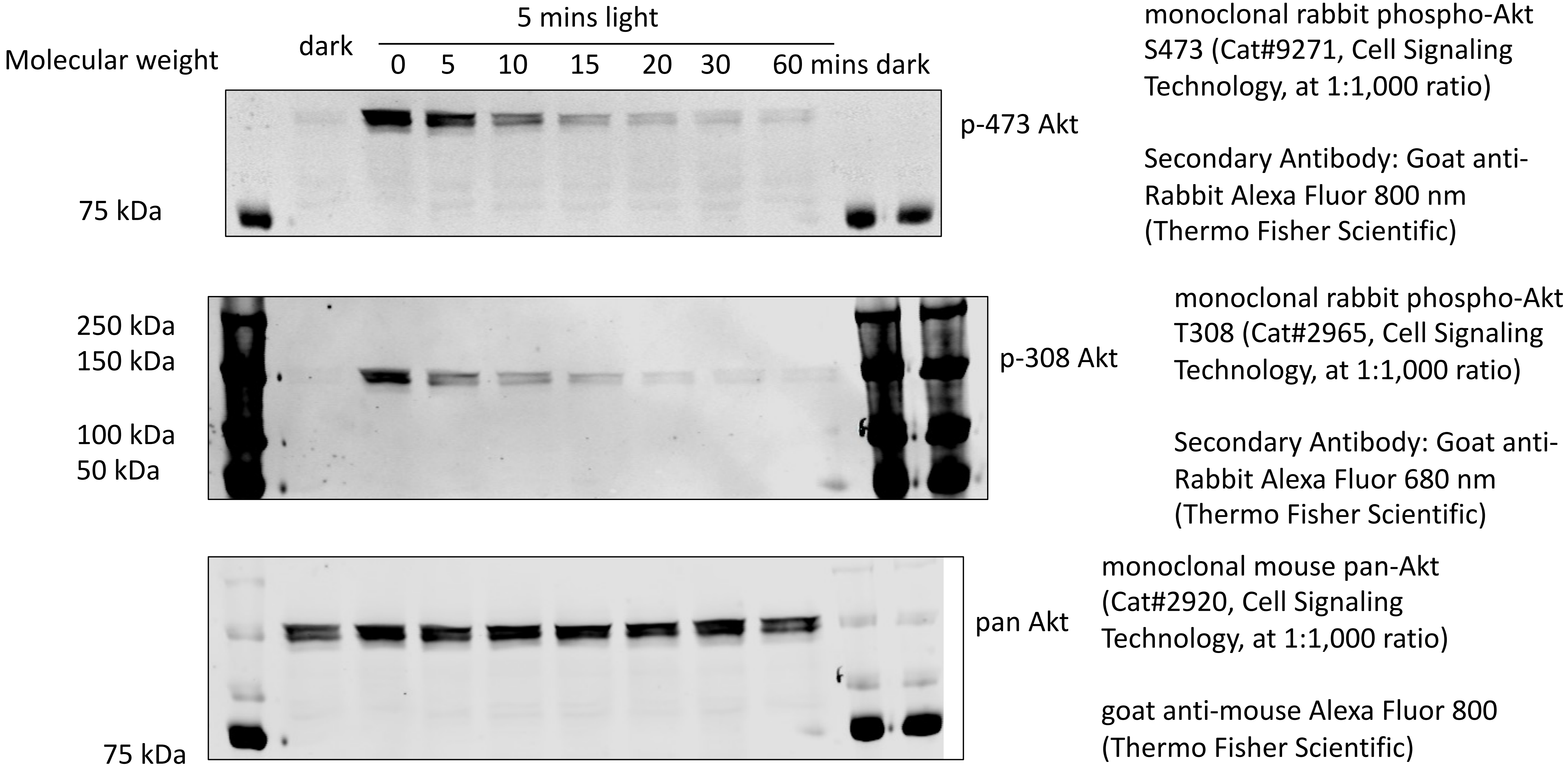
5 mins light
dark
Molecular weight
0
5
10
15
20
30
60 mins dark
75 kDa
p-473 Akt
monoclonal rabbit phospho-Akt S473 (Cat#9271, Cell Signaling Technology, at 1:1,000 ratio)
Secondary Antibody: Goat anti-Rabbit Alexa Fluor 800 nm (Thermo Fisher Scientific)
250 kDa
150 kDa
100 kDa
50 kDa
p-308 Akt
monoclonal rabbit phospho-Akt T308 (Cat#2965, Cell Signaling Technology, at 1:1,000 ratio)
Secondary Antibody: Goat anti-Rabbit Alexa Fluor 680 nm (Thermo Fisher Scientific)
pan Akt
75 kDa
monoclonal mouse pan-Akt (Cat#2920, Cell Signaling Technology, at 1:1,000 ratio)
goat anti-mouse Alexa Fluor 800 (Thermo Fisher Scientific)

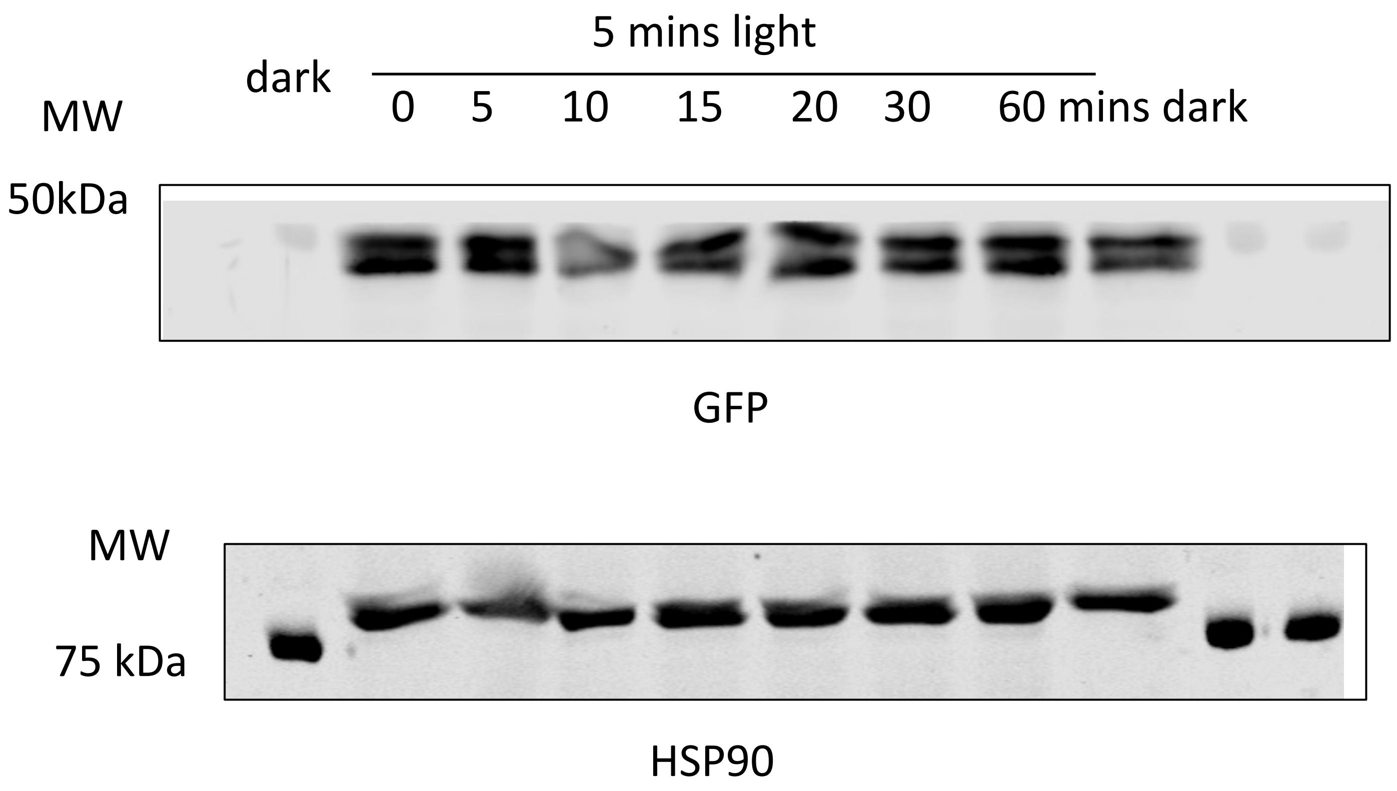

chicken polyclonal anti-GFP (Cat#ab13970, Abcam, at 1:1,000 ratio)

Secondary Antibody: Goat anti-chicken Alexa-Fluor 488 nm (Thermo Fisher Scientific)

monoclonal mouse Hsp90 (Cat#610419, BD Biosciences, at 1:1,000 ratio)

Secondary Antibody: Goat anti-Mouse Alexa-Fluor 800 nm (Thermo Fisher Scientific)

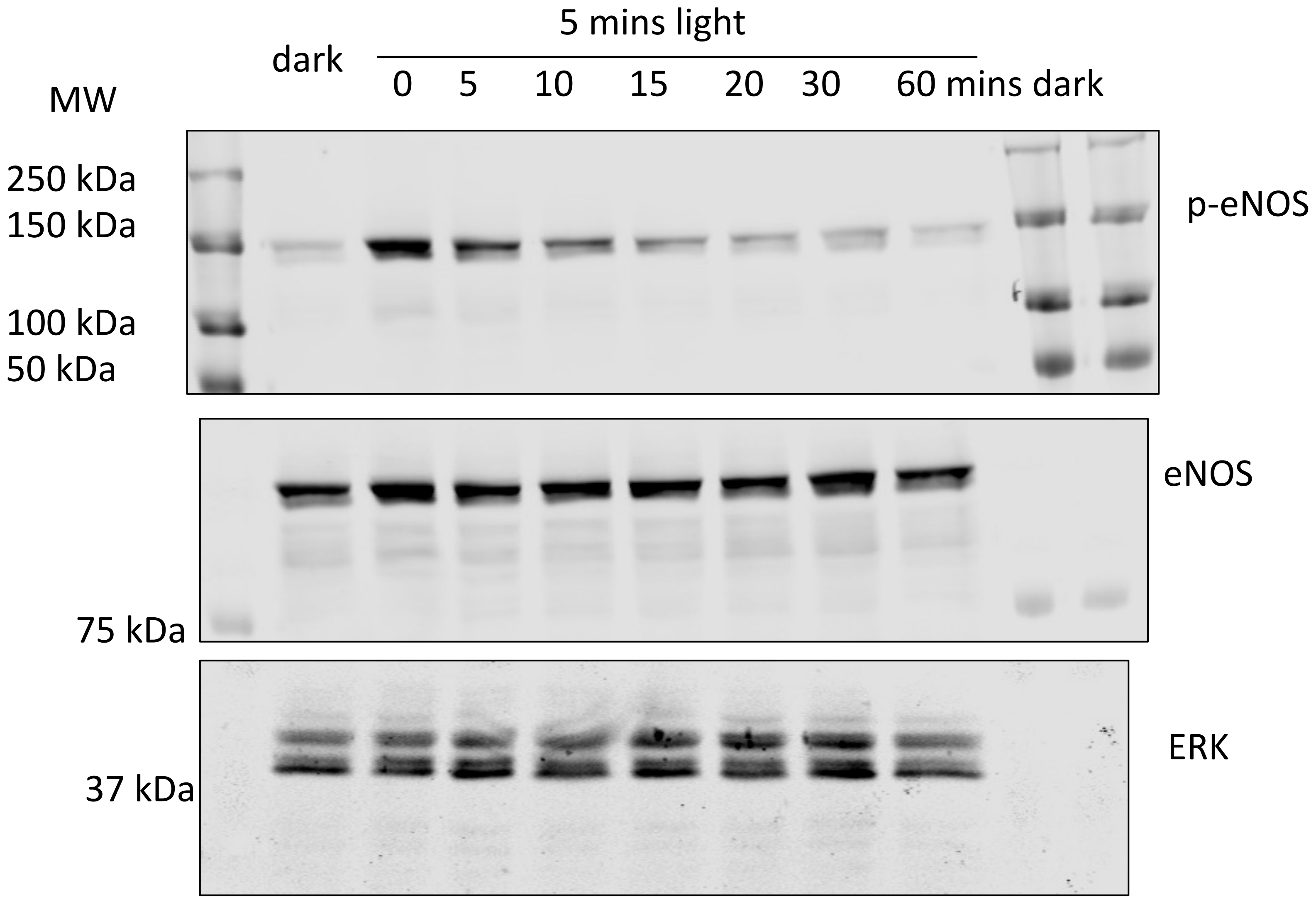

monoclonal rabbit phospho-eNOS S1177 (Cat#9570, Cell Signaling Technology, at 1:1,000 ratio)

Secondary Antibody: Goat anti-Rabbit Alexa-Fluor 680 nm (Thermo Fisher Scientific)

Polyclonal rabbit eNOS (Cat#9572, Cell Signaling Technology, at 1:1,000 ratio)

Secondary Antibody: Goat anti-Rabbit Alexa-Fluor 800 nm (Thermo Fisher Scientific)

monoclonal rabbit Erk(Cat# 4695, cell signaling , at 1:1,000 ratio)

Secondary Antibody: Goat anti-rabbit Alexa-Fluor 800 nm (Thermo Fisher Scientific)

Supplementary Figure 2c

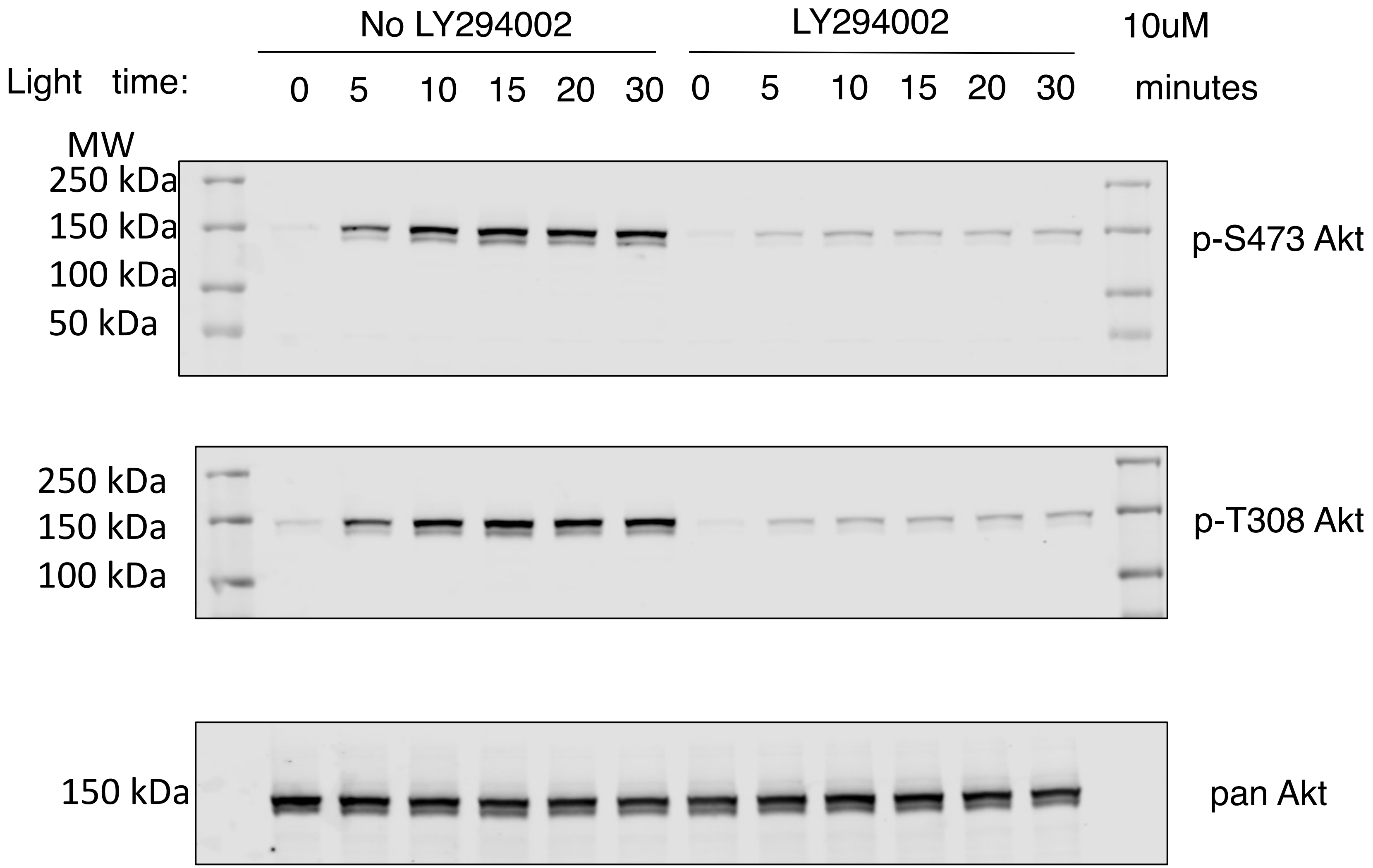

monoclonal rabbit phospho-Akt S473 (Cat#9271, Cell Signaling Technology, at 1:1,000 ratio)

Secondary Antibody: Goat anti-Rabbit Alexa-Fluor 680 nm (Thermo Fisher Scientific)

monoclonal rabbit phospho-Akt T308 (Cat#2965, Cell Signaling Technology, at 1:1,000 ratio)

Secondary Antibody: Goat anti-Rabbit Alexa-Fluor 680 nm (Thermo Fisher Scientific)

monoclonal mouse pan-Akt (Cat#2920, Cell Signaling Technology, at 1:1,000 ratio)

goat anti-mouse Alexa Fluor 800 (Thermo Fisher Scientific)

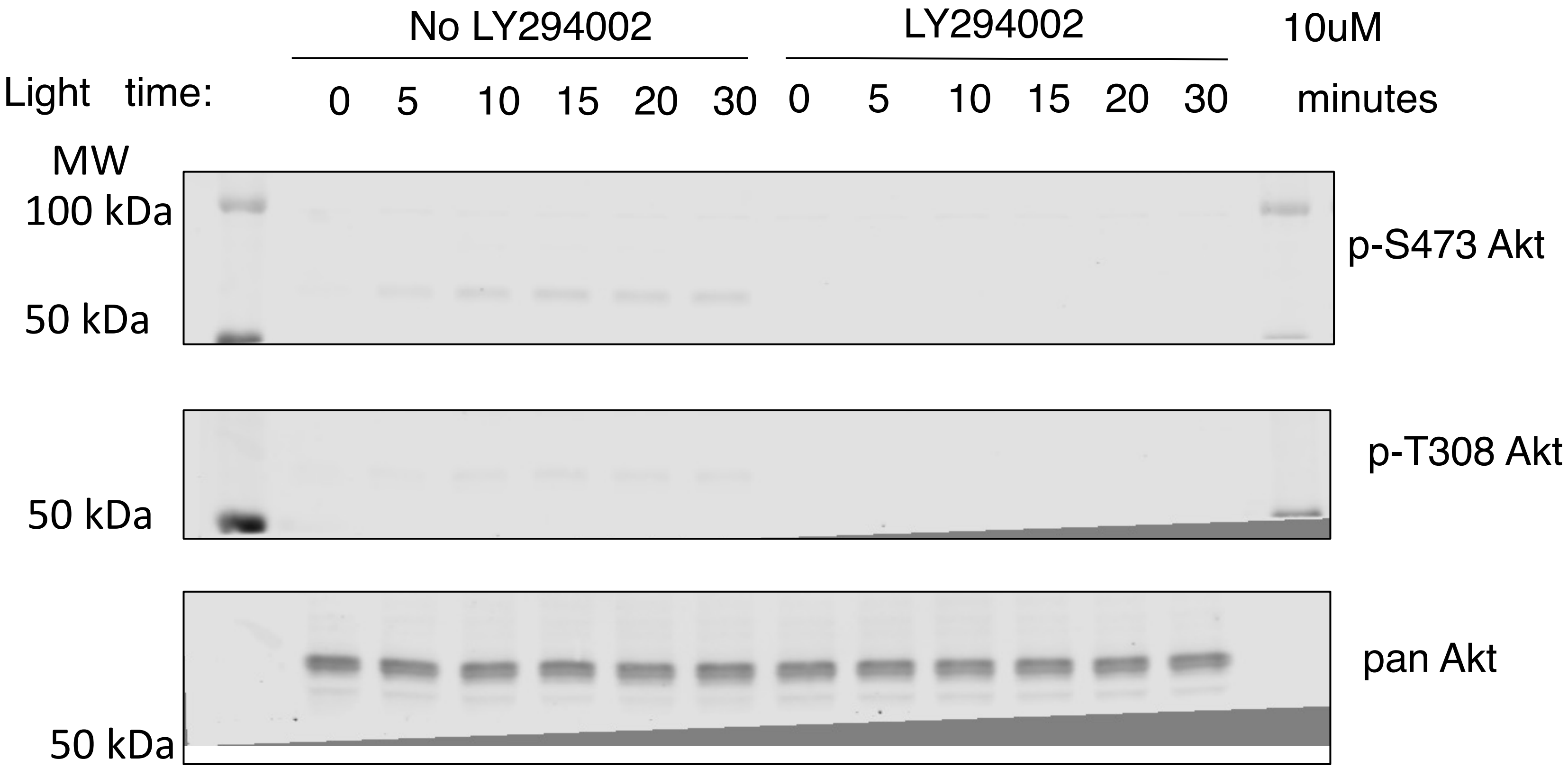

monoclonal rabbit phospho-Akt S473 (Cat#9271, Cell Signaling Technology, at 1:1,000 ratio)

Secondary Antibody: Goat anti-Rabbit Alexa-Fluor 680 nm (Thermo Fisher Scientific)

monoclonal rabbit phospho-Akt T308 (Cat#2965, Cell Signaling Technology, at 1:1,000 ratio)

Secondary Antibody: Goat anti-Rabbit Alexa-Fluor 680 nm (Thermo Fisher Scientific)

monoclonal mouse pan-Akt (Cat#2920, Cell Signaling Technology, at 1:1,000 ratio)

goat anti-mouse Alexa Fluor 800 (Thermo Fisher Scientific)

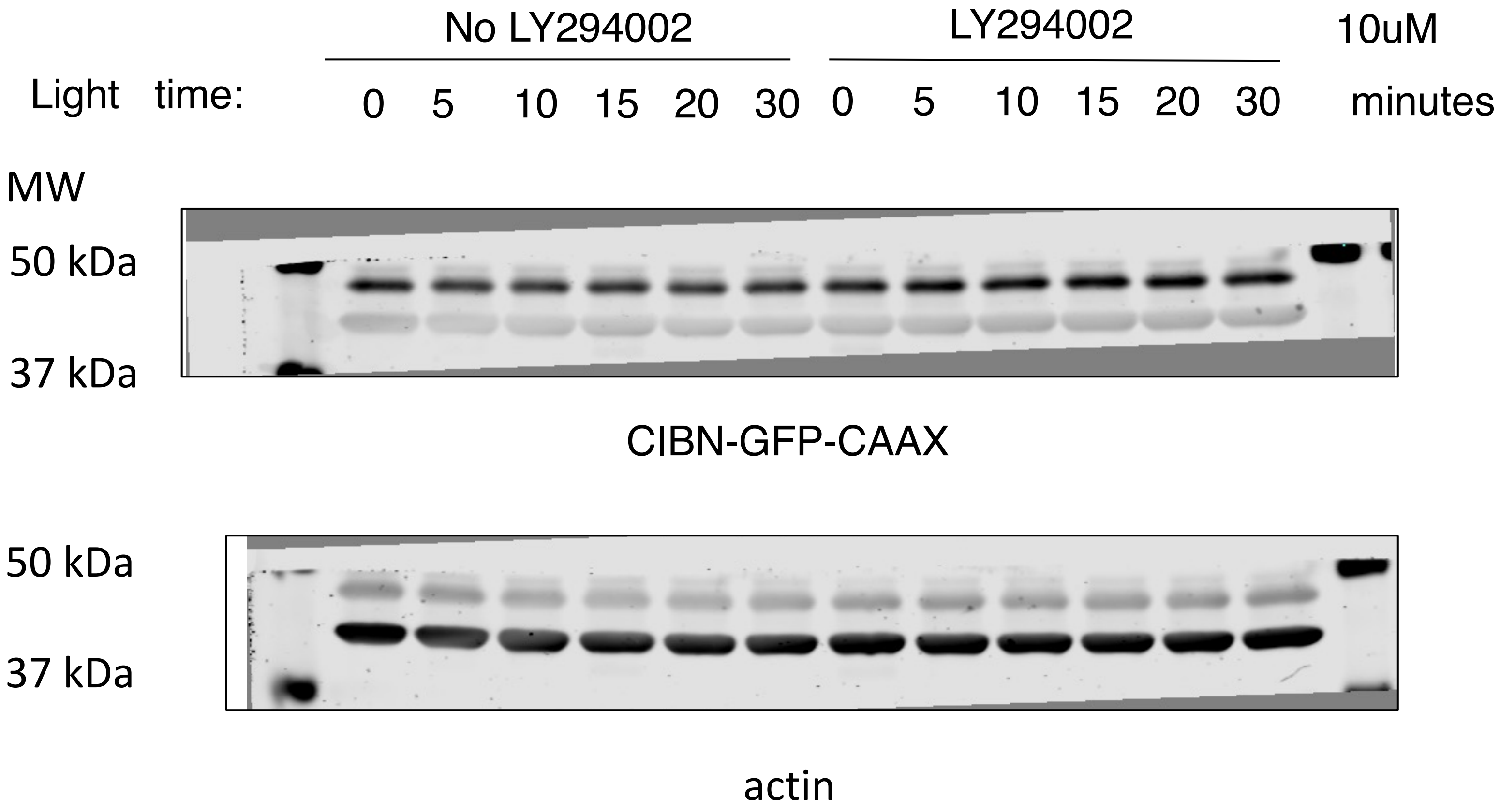

chicken polyclonal anti-GFP (Cat#ab13970, Abcam, at 1:1,000 ratio)

Secondary Antibody: Goat anti-chicken Alexa-Fluor 488 nm (Thermo Fisher Scientific)

monoclonal mouse β-Actin (Cat#A5441, Sigma-Aldrich, at 1:1,000 ratio)

Secondary Antibody: goat anti-mouse Alexa Fluor 680 (Thermo Fisher Scientific)

Supplementary Figure 2d

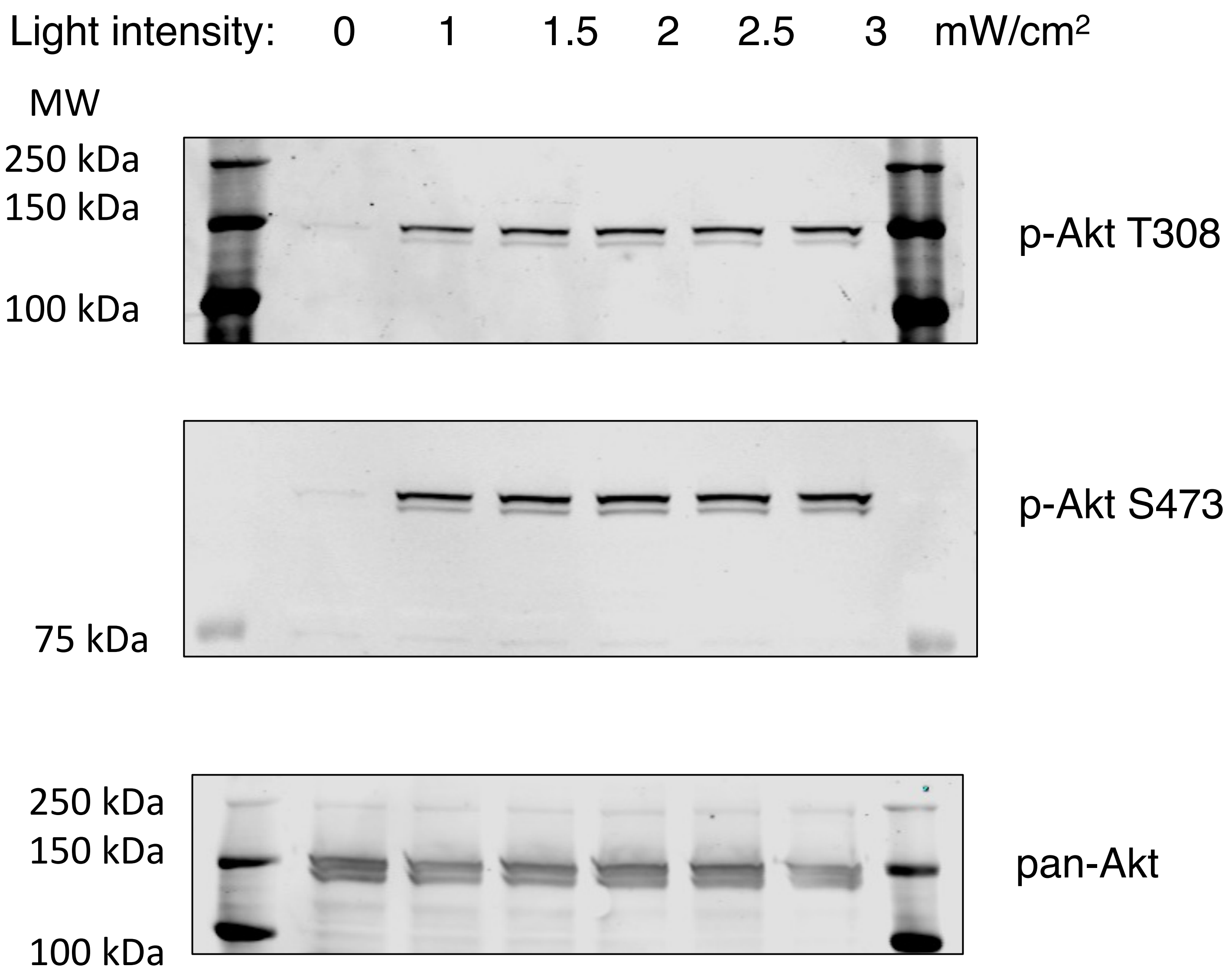

monoclonal rabbit phospho-Akt T308 (Cat#2965, Cell Signaling Technology, at 1:1,000 ratio)

Secondary Antibody: Goat anti-Rabbit Alexa-Fluor 680 nm (Thermo Fisher Scientific)

monoclonal rabbit phospho-Akt S473 (Cat#9271, Cell Signaling Technology, at 1:1,000 ratio)

Secondary Antibody: Goat anti-Rabbit Alexa-Fluor 800 nm (Thermo Fisher Scientific)

monoclonal mouse pan-Akt (Cat#2920, Cell Signaling Technology, at 1:1,000 ratio)

goat anti-mouse Alexa Fluor 680 (Thermo Fisher Scientific)

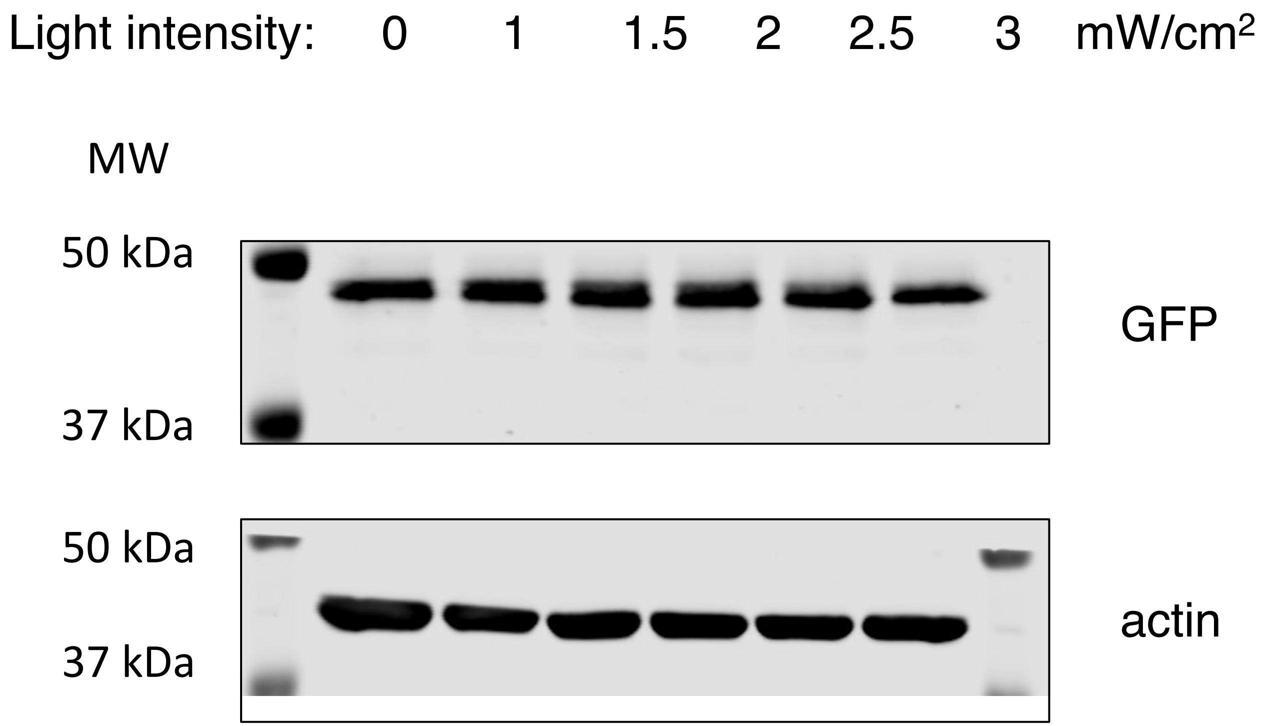

chicken polyclonal anti-GFP (Cat#ab13970, Abcam, at 1:1,000 ratio)

Secondary Antibody: Goat anti-chicken Alexa-Fluor 488 nm (Thermo Fisher Scientific)

monoclonal mouse β-Actin (Cat#A5441, Sigma-Aldrich, at 1:1,000 ratio)

Secondary Antibody: goat anti-mouse Alexa Fluor 680 (Thermo Fisher Scientific)

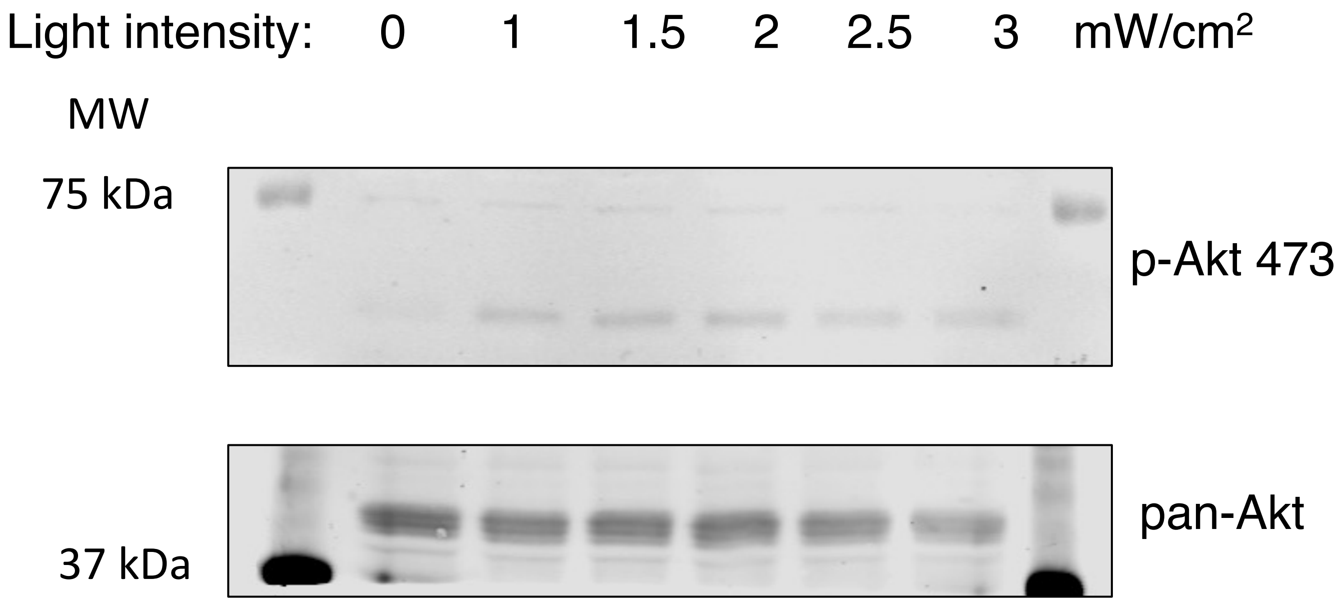

monoclonal rabbit phospho-Akt S473 (Cat#9271, Cell Signaling Technology, at 1:1,000 ratio)

Secondary Antibody: Goat anti-Rabbit Alexa-Fluor 800 nm (Thermo Fisher Scientific)

monoclonal mouse pan-Akt (Cat#2920, Cell Signaling Technology, at 1:1,000 ratio)

goat anti-mouse Alexa Fluor 680 (Thermo Fisher Scientific)

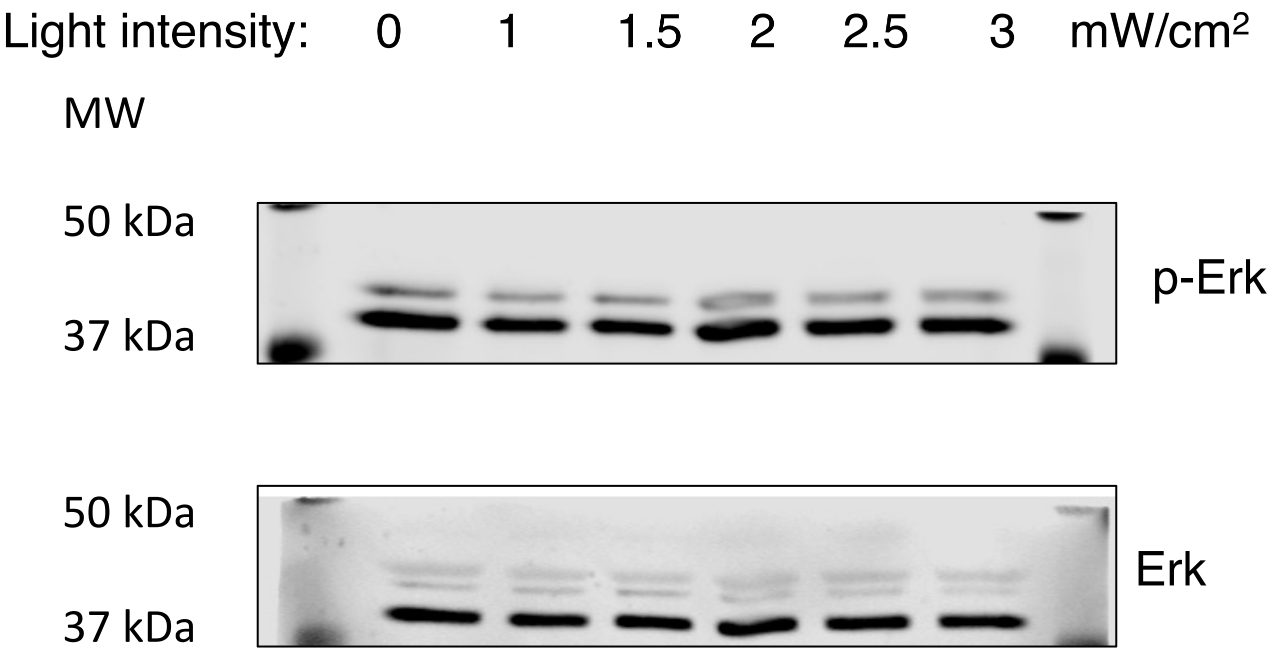

monoclonal rabbit phospho-Erk T202/Tyr204 (Cat#4370 , cell signaling, at 1:1,000 ratio)

Secondary Antibody: Goat anti-rabbit Alexa-Fluor 680 nm (Thermo Fisher Scientific)

monoclonal rabbit Erk(Cat# 4695, cell signaling , at 1:1,000 ratio)

Secondary Antibody: Goat anti-rabbit Alexa-Fluor 680 nm (Thermo Fisher Scientific)

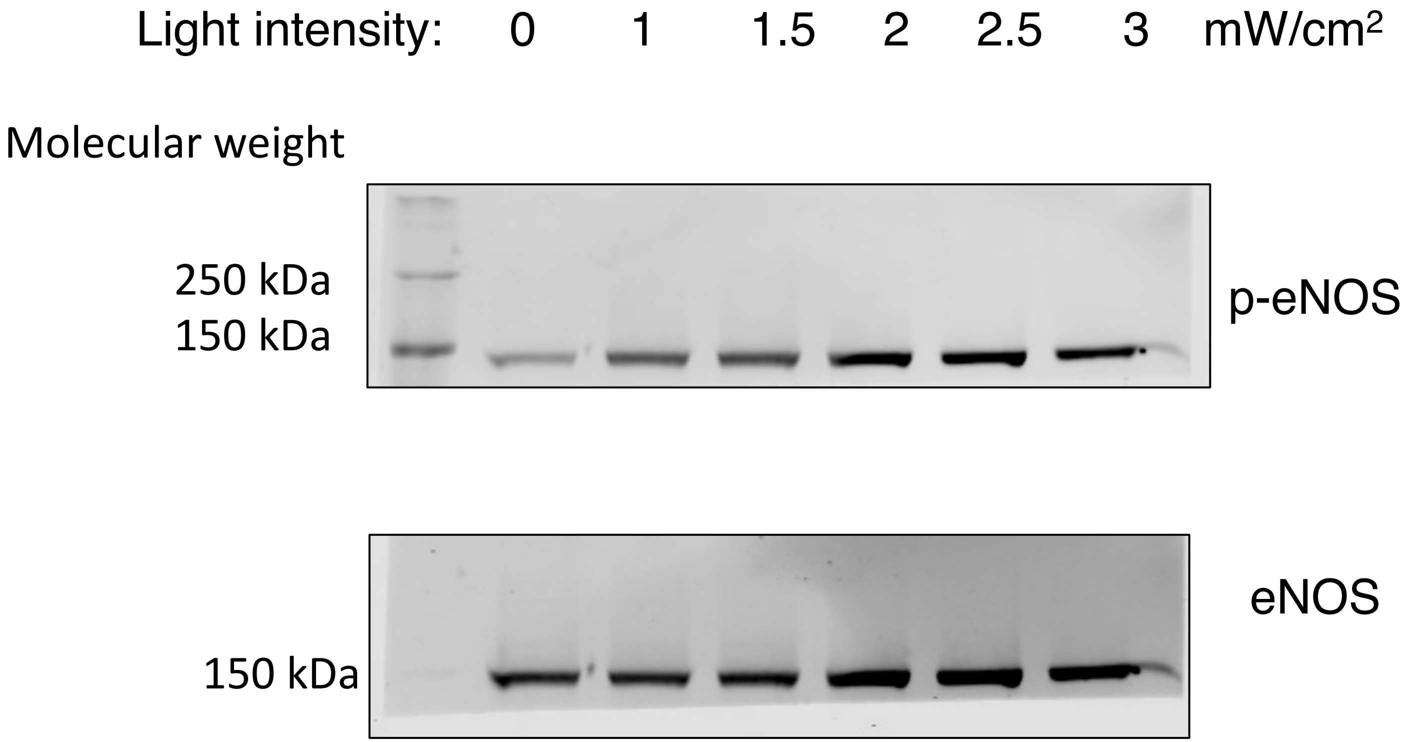

monoclonal rabbit phospho-eNOS S1177 (Cat#9570, Cell Signaling Technology, at 1:1,000 ratio)

Secondary Antibody: Goat anti-Rabbit Alexa-Fluor 680 nm (Thermo Fisher Scientific)

Polyclonal rabbit eNOS (Cat#9572, Cell Signaling Technology, at 1:1,000 ratio)

Secondary Antibody: Goat anti-Rabbit Alexa-Fluor 800 nm (Thermo Fisher Scientific)

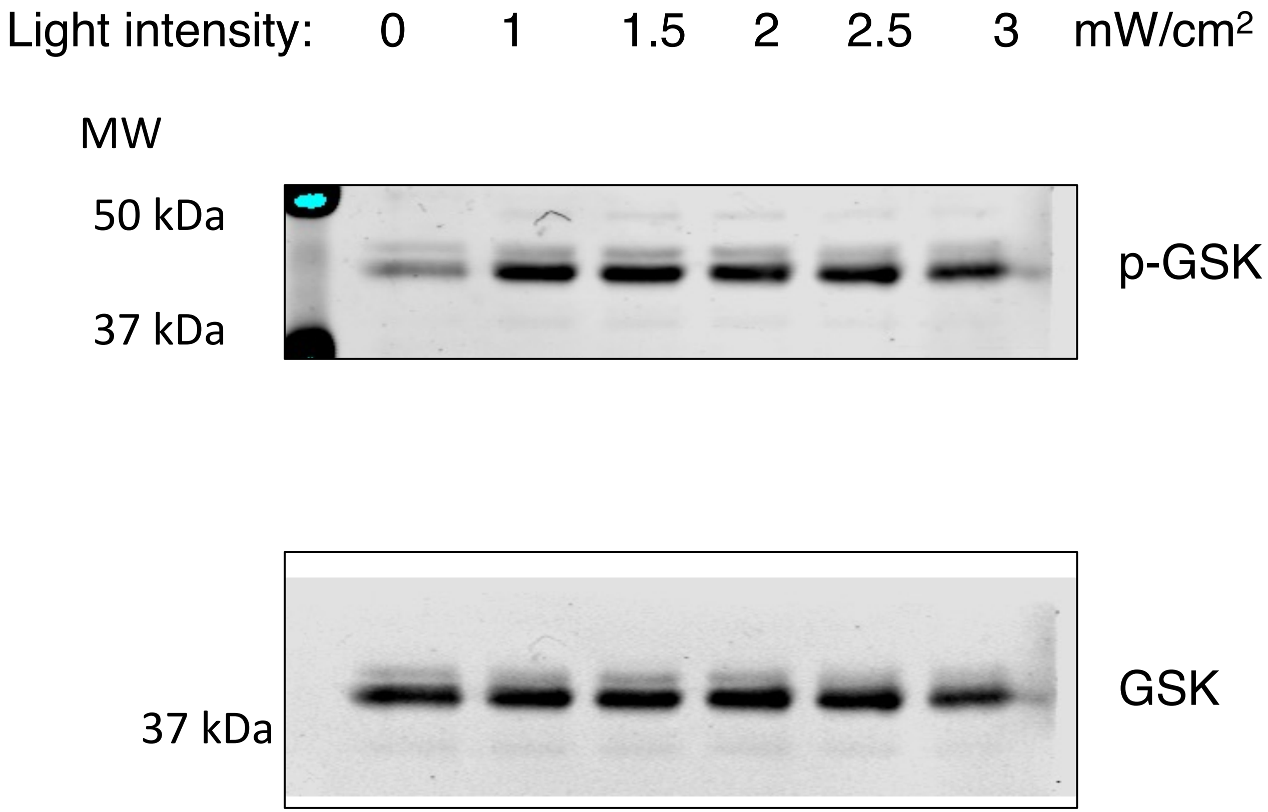

polyclonal rabbit phosphor-GSK-3β S9 (Cat#9336, Cell Signaling Technology, at 1:1,000 ratio)

Secondary Antibody: Goat anti-Rabbit Alexa-Fluor 680 nm (Thermo Fisher Scientific)

polyclonal rabbit phosphor-GSK-3β S9 (Cat#9315, Cell Signaling Technology, at 1:1,000 ratio)

Secondary Antibody: Goat anti-Rabbit Alexa-Fluor 800 nm (Thermo Fisher Scientific)

Supplementary Figure 8a

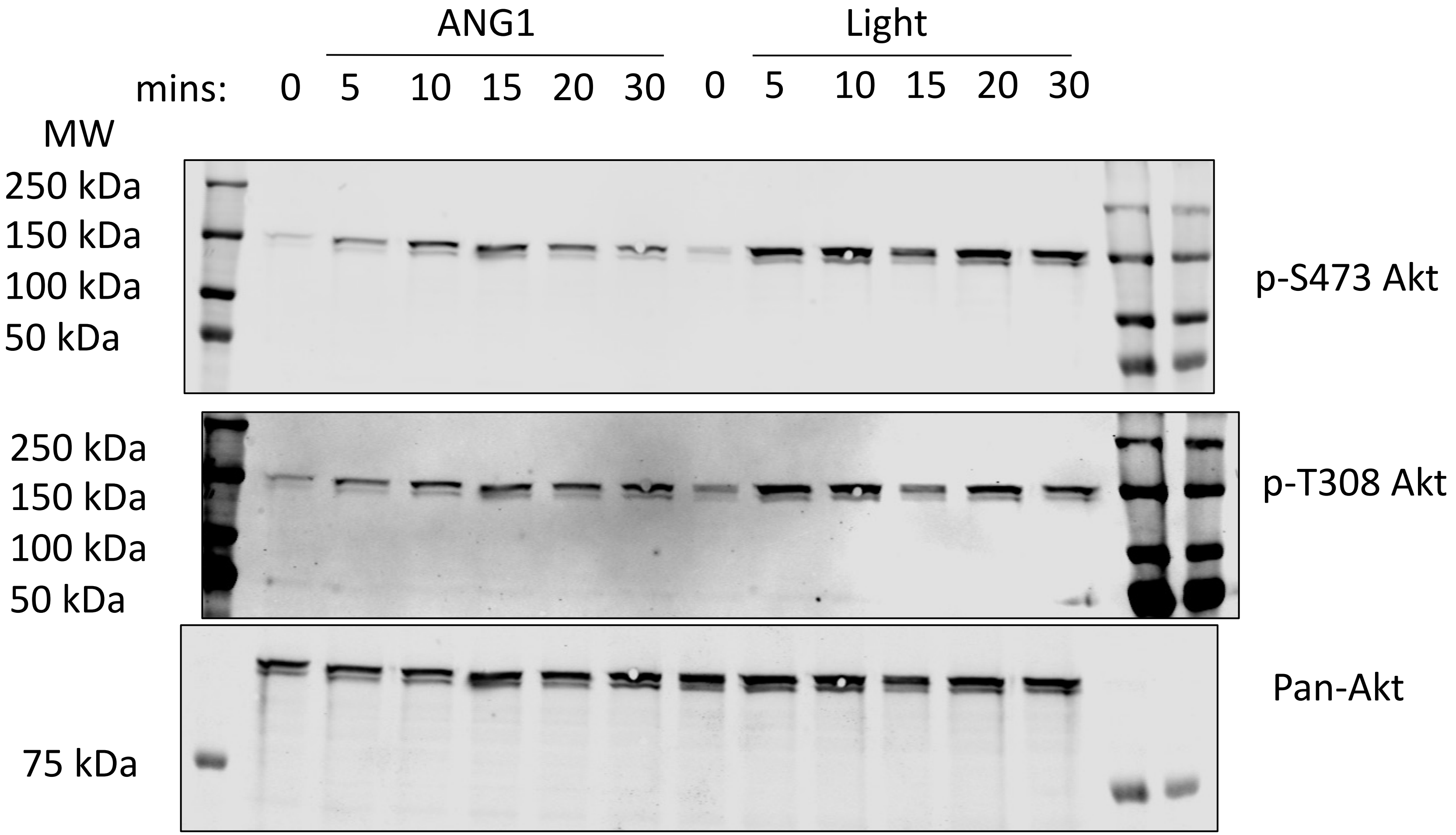

monoclonal rabbit phospho-Akt S473 (Cat#9271, Cell Signaling Technology, at 1:1,000 ratio)

Secondary Antibody: Goat anti-Rabbit Alexa-Fluor 680 nm (Thermo Fisher Scientific)

monoclonal rabbit phospho-Akt T308 (Cat#2965, Cell Signaling Technology, at 1:1,000 ratio)

Secondary Antibody: Goat anti-Rabbit Alexa-Fluor 680 nm (Thermo Fisher Scientific)

monoclonal mouse pan-Akt (Cat#2920, Cell Signaling Technology, at 1:1,000 ratio)

goat anti-mouse Alexa Fluor 800 (Thermo Fisher Scientific)

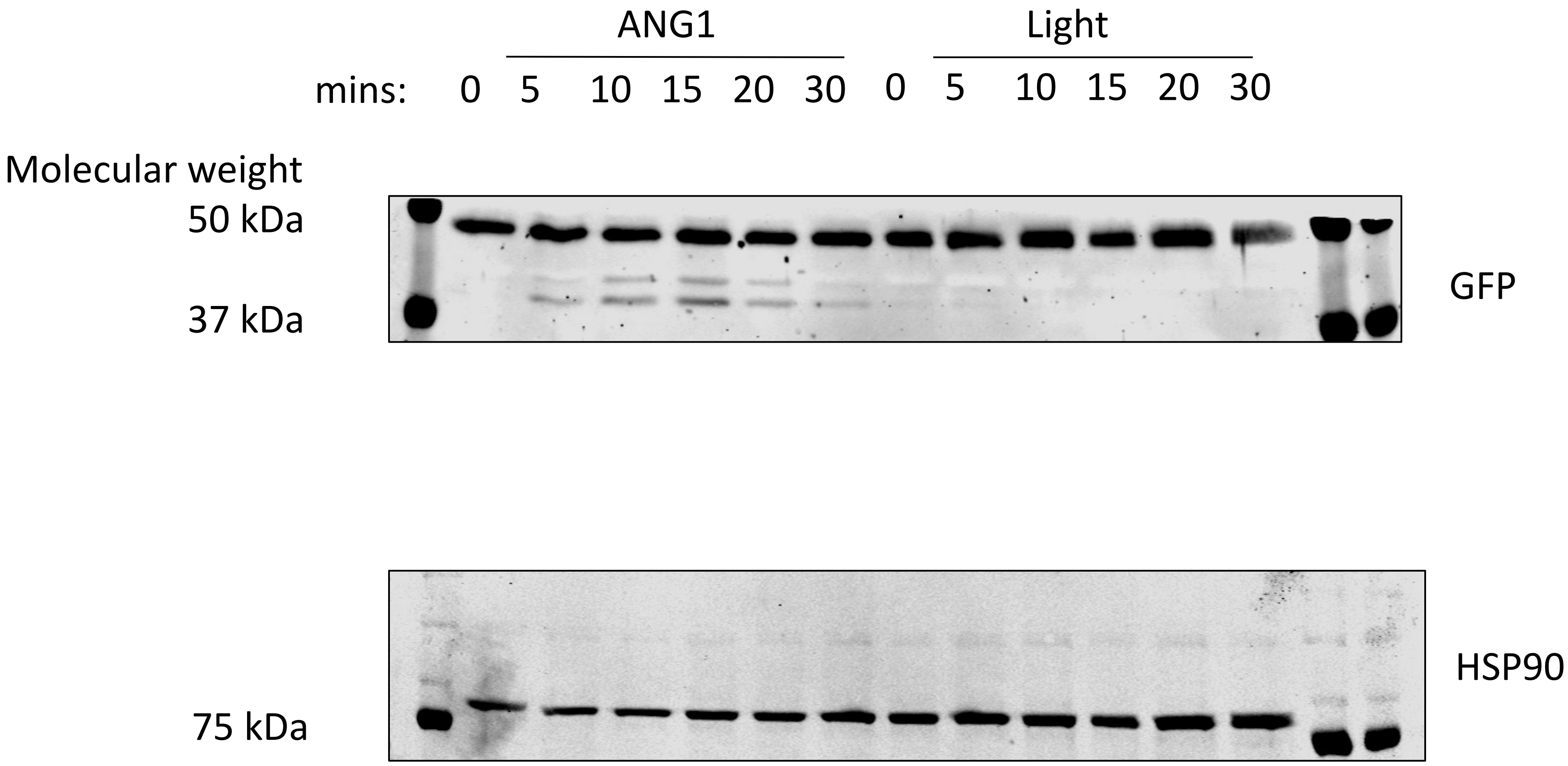

chicken polyclonal anti-GFP (Cat#ab13970, Abcam, at 1:1,000 ratio)

Secondary Antibody: Goat anti-chicken Alexa-Fluor 488 nm (Thermo Fisher Scientific)

monoclonal mouse Hsp90 (Cat#610419 , BD Biosciences, at 1:1,000 ratio)

Secondary Antibody: Goat anti-mouse 800 nm (Thermo Fisher Scientific)

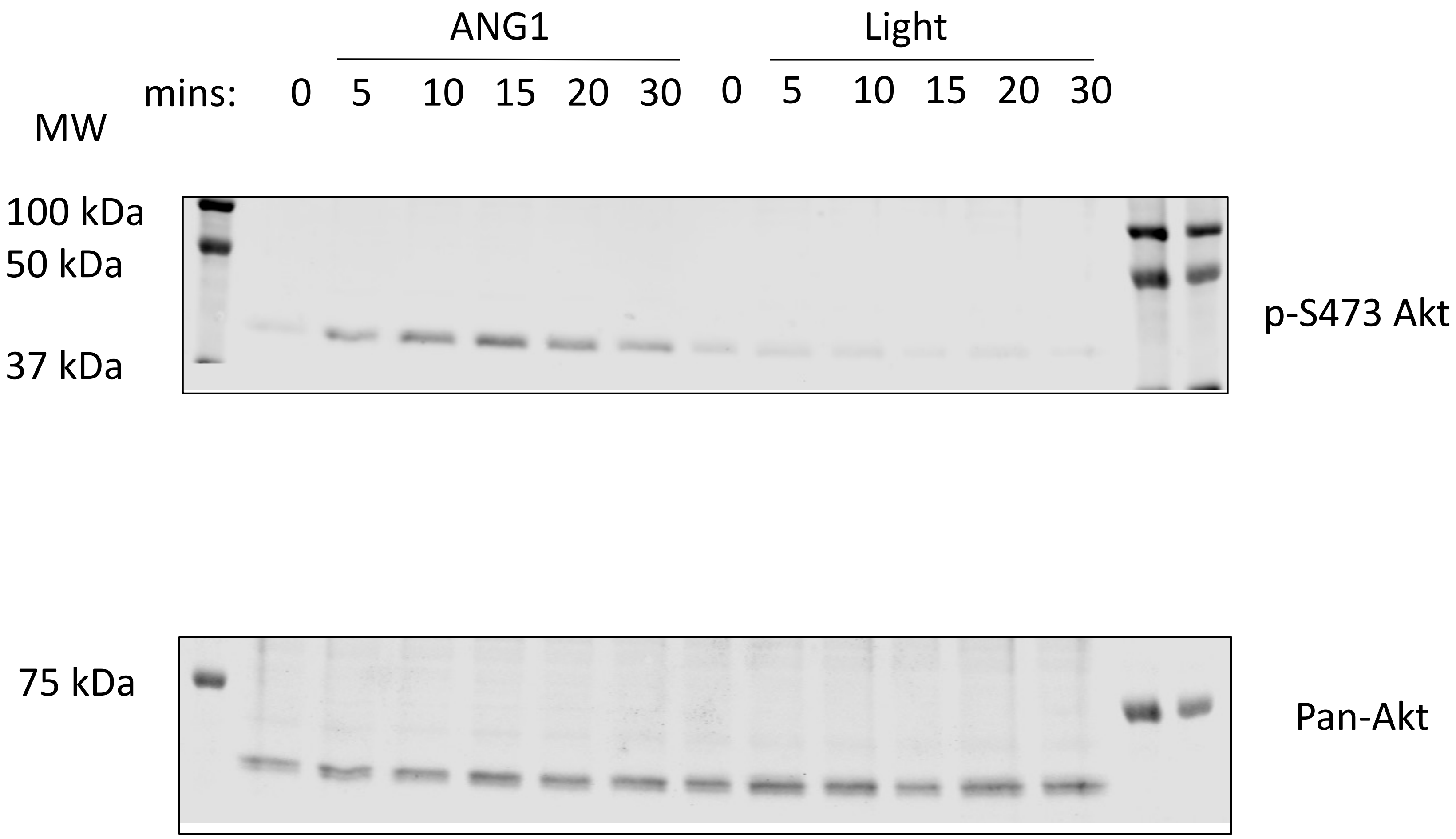

monoclonal rabbit phospho-Akt S473 (Cat#9271, Cell Signaling Technology, at 1:1,000 ratio)

Secondary Antibody: Goat anti-Rabbit Alexa-Fluor 680 nm (Thermo Fisher Scientific)

monoclonal mouse pan-Akt (Cat#2920, Cell Signaling Technology, at 1:1,000 ratio)

goat anti-mouse Alexa Fluor 800 (Thermo Fisher Scientific)

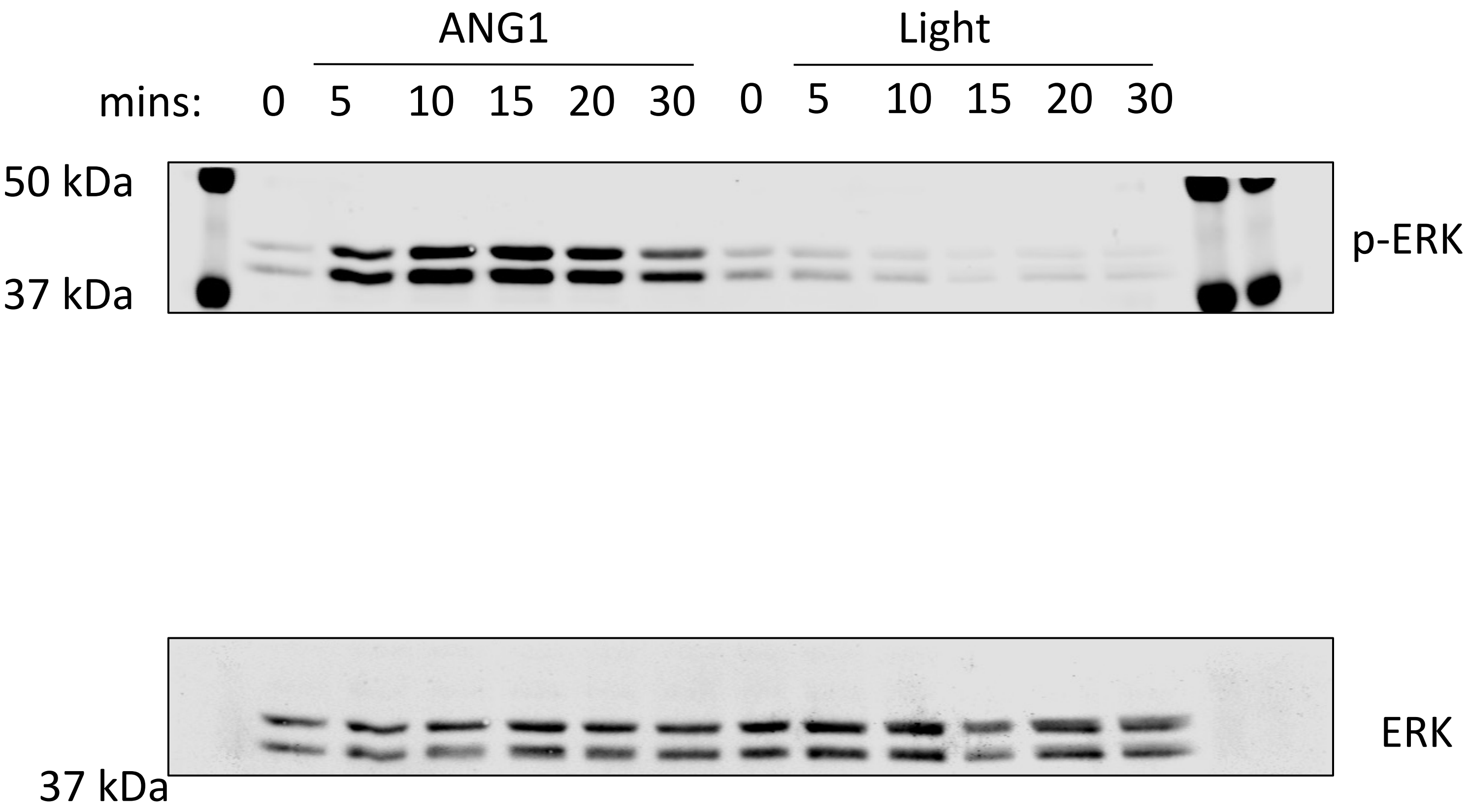

monoclonal rabbit phospho-Erk T202/Tyr204 (Cat#4370 , cell signaling, at 1:1,000 ratio)

Secondary Antibody: Goat anti-rabbit Alexa-Fluor 680 nm (Thermo Fisher Scientific)

monoclonal rabbit Erk(Cat# 4695, cell signaling , at 1:1,000 ratio)

Secondary Antibody: Goat anti-rabbit Alexa-Fluor 800 nm (Thermo Fisher Scientific)
